# Pre-Thrombolysis Biological Correlates of Admission Glasgow Coma Scale in Older Adults with Acute Ischemic Stroke: A Retrospective Single-Center Cohort Study

**DOI:** 10.3390/medicina62091692

**Published:** 2026-09-03

**Authors:** Sorina Nicoleta Munteanu, Adrian Stan, Aurel Nechita, Dorel Firescu, Mihai Cristian Marinescu, Claudiu Elisei Tanase, Ioana Navalici, Dana Tutunaru, Mihaela Moisei, Aurelia Romila

**Affiliations:** 1 Research Centre in the Medical-Pharmaceutical Field, Faculty of Medicine and Pharmacy, “Dunarea de Jos” University of Galati, 800008 Galati, Romania; sorina.munteanu@ugal.ro (S.N.M.); aurel.nechita@ugal.ro (A.N.); dorel.firescu@ugal.ro (D.F.); cardiologmihaimarinescu@gmail.com (M.C.M.); tanaseclaudiumd@gmail.com (C.E.T.); navalio@gmail.com (I.N.); dana.tutunaru@ugal.ro (D.T.); aurelia.romila@yahoo.com (A.R.); 2 “Saint Andrew the Apostle” Clinical Emergency County Hospital, 800578 Galati, Romania; 3 “Saint John” Clinical Emergency Hospital for Children, 800487 Galati, Romania

**Keywords:** acute ischemic stroke, Glasgow Coma Scale, intravenous thrombolysis, anemia, iron status, ferritin, vitamin B12, C-reactive protein, red cell distribution width, mean corpuscular volume

## Abstract

*Background and Objectives*: Biological correlates of baseline clinical responsiveness remain incompletely characterized in older adults with acute ischemic stroke treated with intravenous thrombolysis. We evaluated associations between admission Glasgow Coma Scale (GCS) and pre-thrombolysis hematologic, inflammatory, iron-status, and micronutrient parameters. *Materials and Methods*: This retrospective single-center cohort included 95 unique patients aged ≥65 years, all treated with intravenous alteplase between 2020 and 2024. Admission GCS ranged from 11 to 14. Spearman correlations used original GCS scores. Principal proportional odds models used ordered GCS categories of 11–12, 13, and 14, and were adjusted for age, sex, and admission National Institutes of Health Stroke Scale (NIHSS), with false discovery rate corrections. Sensitivity analyses additionally considered clinician-documented aphasia, congestive heart failure, atrial fibrillation, and reperfusion status for 24 h GCS change. *Results*: Admission GCS correlated most strongly with RDW-CV (rho = −0.843; 95% bootstrap CI, −0.892 to −0.774) and MCV (rho = 0.779; 95% CI, 0.668 to 0.866). Principal adjusted odds ratios per one-SD increase were 5.68 for hemoglobin, 3.01 for log-transformed ferritin, 4.71 for serum iron, 4.71 for log-transformed vitamin B12, 0.20 for C-reactive protein, and 0.019 for RDW-CV (all FDR-adjusted *p* < 0.001). Association directions remained consistent in sensitivity analyses. MCV showed an exploratory nonlinear association. No biomarker was associated with the direction of 24 h GCS change after FDR correction, including reperfusion-adjusted models. *Conclusions*: Pre-thrombolysis biological parameters were concurrently associated with baseline GCS within this single-center cohort and restricted GCS range. These associations do not establish causality, independence from unmeasured lesion location or infarct volume, predictive utility, or early neurological recovery. Prospective external validation is required.

## 1. Introduction

Acute ischemic stroke in older adults requires rapid integration of neurological findings, comorbidity, and laboratory data at presentation. Although intravenous thrombolysis improves outcomes in eligible patients, initial clinical responsiveness and subsequent trajectories remain heterogeneous.

The Glasgow Coma Scale (GCS) provides a standardized assessment of ocular, verbal, and motor responsiveness. Reduced responsiveness in acute ischemic stroke has been associated with greater clinical severity and adverse outcomes [1,2]. In the present study, admission GCS was selected as the principal baseline neurological measure because the research question concerned concurrent pre-thrombolysis biological correlates of overall responsiveness at emergency presentation rather than a post-treatment functional endpoint. GCS and the National Institutes of Health Stroke Scale (NIHSS) assess overlapping but non-equivalent constructs: NIHSS emphasizes focal neurological deficits, whereas GCS summarizes overall ocular, verbal, and motor responsiveness. GCS is not stroke-specific, however, and its total scores may be influenced by aphasia, dysarthria, motor deficits, impaired comprehension, and lesion characteristics. It was therefore not treated as a substitute for NIHSS, lesion-based assessments, or conventional functional outcomes.

Hematologic status may influence the clinical presentation of cerebral ischemia through oxygen transport, blood rheology, erythrocyte morphology, inflammation, and nutritional status. Hemoglobin has shown nonlinear or inconsistent associations with stroke outcomes [3,4], whereas a higher red cell distribution width (RDW) has been associated with greater neurological severity and unfavorable outcome [5]. Mean corpuscular volume (MCV) provides complementary information and may itself have a nonlinear relationship with post-stroke outcomes [6].

Iron availability, vitamin B12 status, and systemic inflammation may provide additional information. Iron deficiency at admission has been associated with unfavorable three-month outcomes [7], lower vitamin B12 concentrations with stroke severity and functional outcomes [8], and higher C-reactive protein (CRP) with unfavorable outcomes and mortality after intravenous thrombolysis [9]. Ferritin is also an acute-phase reactant while CRP is nonspecific; these measures require interpretation within an integrated hematologic and inflammatory profile.

Evidence remains limited regarding the integrated relationships between pre-thrombolysis hematologic, inflammatory, iron-status, and micronutrient parameters and overall admission responsiveness in thrombolysis-treated older adults. Previous studies have generally focused on NIHSS, mortality, or modified Rankin Scale outcomes and have often evaluated individual biomarkers rather than coordinated biological domains.

The primary objective was to evaluate associations between admission GCS and pre-thrombolysis hemoglobin, RDW-CV, MCV, ferritin, serum iron, vitamin B12, and CRP in adults aged 65 years or older with acute ischemic stroke treated with intravenous alteplase.

Principal adjusted analyses accounted for age, sex, and admission NIHSS. Secondary objectives were to describe the distribution of clinically defined anemia, low ferritin, and low vitamin B12 statuses across admission GCS categories; to evaluate the stability of the principal associations in targeted analyses, accounting for clinician-documented aphasia and prominent cardiovascular comorbidities; and to explore GCS at 24 h and the direction of early GCS change.

We hypothesized that higher hemoglobin, iron, ferritin, and vitamin B12 and lower RDW-CV and CRP would be associated with higher admission GCS categories; no uniform linear direction was prespecified for MCV.

## 2. Materials and Methods

### 2.1. Study Design and Setting

This retrospective, single-center, observational cohort study analyzed index-hospitalization records of adults aged 65 years or older with acute ischemic stroke who received intravenous alteplase at the “Saint Andrew the Apostle” Clinical Emergency County Hospital, Galati, Romania, between 2020 and 2024.

The study analyzed routinely collected clinical data. No research-specific examinations, patient contact, or treatment modifications occurred.

The study was reported in accordance with the Strengthening the Reporting of Observational Studies in Epidemiology (STROBE) recommendations for cohort studies.

### 2.2. Source Population and Cohort Derivation

The institutional source database comprised 200 hospitalization records for acute ischemic stroke during 2020–2024. This number represented hospitalization episodes rather than 200 unique individuals. Sixty hospitalization records involved patients younger than 65 years and were excluded, leaving 140 age-eligible hospitalization records.

Intravenous thrombolysis had not been administered in 42 of the 140 age-eligible hospitalization records. These records were excluded, leaving 98 hospitalization records that met both the age and thrombolysis treatment criteria. Selection at this stage was based on eligibility characteristics rather than on admission GCSs, biomarker values, subsequent neurological evolution, or clinical outcomes.

The 98 eligible hospitalization records were subsequently evaluated at the patient level to identify repeated eligible admissions. Three records represented later eligible hospitalizations of individuals who already had an earlier hospitalization meeting the study criteria.

Patient-level deduplication was therefore performed by identifying records belonging to the same individual and retaining only the earliest eligible hospitalization as the index hospitalization. Removal of the three later eligible admissions resulted in a final cohort of 95 unique patients.

The analytical unit was the individual patient rather than the hospitalization episode. Each included individual therefore contributed only one index hospitalization to the analyses. This patient-level approach prevented repeated eligible admissions from contributing multiple observations to the same analytical cohort and preserved a single baseline clinical and biological assessment for each participant.

All 95 retained patients had complete data for admission GCS, the principal biological exposures, age, sex, and admission NIHSS. The entire deduplicated eligible cohort was analyzed.

No prospective sample-size calculation or sampling procedure was performed because the study retrospectively included all unique patients who fulfilled the predefined eligibility criteria during the study period. No patient was excluded on the basis of biomarker concentration, admission GCS magnitude or category, bleeding status, 24 h neurological evolution, length of hospitalization, or in-hospital outcome.

Thus, cohort construction proceeded from hospitalization-level screening to patient-level deduplication before statistical analysis. This distinction was maintained throughout the study to ensure that the reported sample size referred to unique individuals rather than hospitalization episodes.

**Figure 1:**
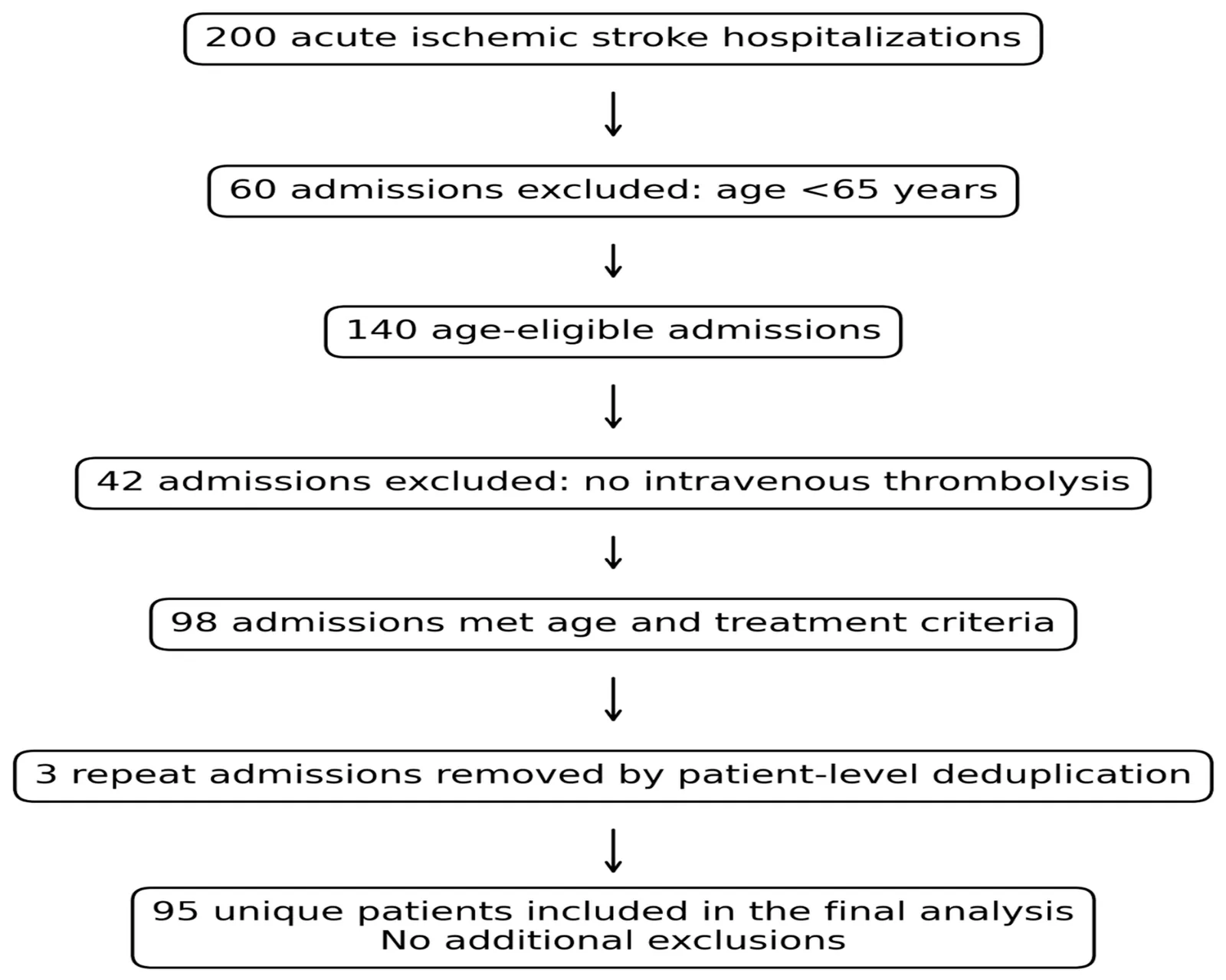
Cohort derivation. Arrows indicate the sequential flow from the source hospitalization records through eligibility assessment and patient-level deduplication to the final analytical cohort.

The complete sequence of record screening, eligibility assessment, deduplication, and derivation of the final 95-patient analytical cohort is summarized in Figure 1.

The same source cohort is also analyzed in a companion manuscript entitled “Hospitalization-Documented Gingival Bleeding, Hematuria, and Upper Gastrointestinal Bleeding in Older Adults Treated With Intravenous Alteplase for Acute Ischemic Stroke: A Retrospective Single-Center Cohort Study”.

The present study addresses a distinct question: admission GCS is the principal baseline dependent neurological measure, and pre-thrombolysis biological variables are the principal exposures.. No bleeding-status comparisons, site-specific bleeding results, or hemorrhagic-outcome models are included here. The research questions, analytical endpoints, analyses, tables, figures, and conclusions are distinct between the two manuscripts.

### 2.3. Eligibility Criteria

Patients were eligible if they were aged 65 years or older at the index hospitalization; had a treating-team diagnosis of acute ischemic stroke; received intravenous alteplase; and had complete data for the principal hematologic, inflammatory, iron-status, and micronutrient measurements, admission GCS, and admission NIHSS, together with sufficient information for patient-level deduplication.

Patients were excluded if they were younger than 65 years, had not received intravenous alteplase, represented a later eligible hospitalization after the earliest retained index hospitalization, or lacked admission GCS or principal biological exposure data. No exclusion was based on a particular GCS value, and no exclusions attributable to missing principal data occurred after patient-level deduplication.

### 2.4. Data Collection and Timing of Measurements

For clarity, the study design, source-population derivation, and eligibility criteria are described in Section 2.1, Section 2.2 and Section 2.3. The present section consolidates the methodological details regarding source-data abstraction and patient-level record linkage, the clinical and neuroimaging basis of the acute ischemic stroke diagnosis, the availability and limitations of imaging information, the retained vascular-territory and reperfusion classifications, intravenous thrombolysis treatment, and the availability of mechanical thrombectomy data.

Demographic characteristics, medical history, pre-stroke medication, neurological assessments, laboratory results, treatment-related information, and in-hospital outcomes were abstracted from the hospital electronic information system and associated routine-care source documentation for the retained index hospitalization. The data-curation workbook used a pseudonymized Study_ID for analytical linkage and retained the source medical record number only for internal source verification. A concordance audit confirmed matching Study_IDs and source record numbers for all 95 patients between the principal analytical dataset and the linked stroke-characterization table. The source record number was not treated as an analytical variable or reported in patient-level study outputs. Names, personal identification numbers, contact details, and other direct identifiers were excluded from the analytical dataset.

Blood samples for hemoglobin, RDW-CV, MCV, serum ferritin, serum iron, vitamin B12, and CRP were obtained during the initial emergency assessment after hospital presentation and before the alteplase bolus. These measurements therefore characterized the pre-thrombolysis biological profile used in the principal analyses. The retained dataset did not preserve a standardized exact interval between each blood draw and alteplase administration. Manufacturer and model information for the routine-care laboratory analyzers and reagents was not retained in the retrospective analytical archive and therefore could not be reported reliably.

Acute ischemic stroke was diagnosed by the treating stroke team on the basis of an acute neurological deficit judged clinically compatible with cerebral ischemia, combined with emergency neuroimaging used to exclude intracranial hemorrhage and assess eligibility for reperfusion. A patient-linked stroke-characterization table was available for the same 95 individuals after the concordance of Study_IDs and source record numbers was verified. Broad vascular territory and routine-care complete/partial reperfusion fields were available for all 95 patients, whereas ASPECTS was recorded in only six linked records. The archived data did not contain a standardized patient-level field specifying non-contrast CT, CT angiography, CT perfusion, or MRI for every case, and did not provide uniform lesion-volume or collateral-status variables. Accordingly, imaging-modality-specific, ASPECTS-based, lesion-volume, and collateral analyses were not performed.

The retained archive did not allow uniform retrospective determination of whether an acute ischemic lesion was directly visualized via initial neuroimaging for every included patient. Accordingly, the study does not claim imaging-based confirmation of cerebral infarction in all 95 patients; the operational diagnosis reflects the treating stroke team’s clinical diagnosis that was supported by emergency neuroimaging used primarily to exclude intracranial hemorrhage and guide acute reperfusion eligibility.

The linked stroke-characterization table retained only broad routine-care vascular-territory labels—carotid, vertebrobasilar, or undetermined. Detailed vessel-level or anatomical criteria used to assign these labels were not retained, and the categories were therefore reported as recorded without retrospective reclassification.

The complete and partial reperfusion fields likewise reflected routine-care documentation. The archive did not retain a uniform Thrombolysis in Cerebral Infarction (TICI) or modified TICI (mTICI) grade, nor did it retain a standardized patient-level angiographic adjudication rule defining complete versus partial reperfusion. These categories were therefore treated as descriptive routine-care labels rather than validated angiographic endpoints.

The corresponding patient-linked distributions of broad vascular territories, documented complete or partial reperfusions, and ASPECTS availability are summarized in Supplementary Table S7.

Eligibility for intravenous thrombolysis was determined by the treating stroke team under the local acute stroke protocol in force at the time of treatment. A separate 95-record clinical process table retained pre-treatment systolic and diastolic blood pressure, platelet counts, INR, aPTT, creatinine, onset-to-needle times, and door-to-needle times. This table did not contain Study_IDs, and its row order did not match the patient-level analytical dataset; therefore, it was not merged row by row into the GCS–biomarker analyses. The study archive did not retain protocol-version metadata sufficient to attribute each case to a specific European Stroke Organisation, AHA/ASA, or other external guideline edition. All 95 patients in the analytical cohort received intravenous alteplase; none were classified as receiving tenecteplase. Alteplase was administered at 0.9 mg/kg, with 10% of the total dose given as an initial bolus and the remaining 90% infused over 60 min.

Mechanical thrombectomy status and the patient-level criteria used to determine eligibility for endovascular therapy were not retained as uniform linkable variables in the available analytical archive. A reliable proportion of patients undergoing mechanical thrombectomy and the corresponding eligibility criteria therefore could not be reported or inferred retrospectively. Mechanical thrombectomy was not used as an analytical exposure or covariate in this study.

Admission GCS and the standard total NIHSS score were recorded by the attending neurologist during the same initial pre-thrombolysis neurological evaluation before alteplase administration. The analytical dataset retained only total GCS scores and did not preserve the separate eye, verbal, and motor component scores; component-level GCS analyses were therefore not possible. Aphasia was extracted as a binary clinician-documented finding from the same routine-care admission neurological assessment. Aphasia was not assessed using a research-specific standardized language battery and was not independently adjudicated against a second reference assessment. Consequently, false positive and false negative aphasia rates could not be estimated from the retained data. The aphasia variable was used for descriptive cohort characterization and as a covariate in a targeted sensitivity analysis, but it was not treated as a validated research-grade language measure.

### 2.5. Principal Baseline Neurological Measure and Exploratory 24 h Measures

The principal dependent neurological measure was admission GCS, recorded before thrombolysis as a baseline neurological assessment rather than as a follow-up clinical outcome. Patient-level re-verification of the final analytical database confirmed the observed distribution of GCS 11 in 2 patients, GCS 12 in 19, GCS 13 in 35, and GCS 14 in 39; no patient had an admission GCS 15. The 11–14 interval was the observed range of the cohort and was not an eligibility criterion. Original total scores were retained for descriptive analyses and Spearman rank correlations.

For proportional odds ordinal regression, admission GCS was represented by three ordered categories: GCS 11–12, GCS 13, and GCS 14. The two patients with GCS 11 were combined with the immediately adjacent GCS 12 category to avoid fitting a separate outcome stratum containing only two observations, which would increase coefficient instability and sparse-data sensitivity. This grouping was an analytical decision for the ordinal models and not a clinical cutoff; the original four-level scores remained the basis of the rank correlation analyses.

Exploratory neurological measures included GCS at 24 h and the change in GCS during the first 24 h. Change was defined a priori as GCS at 24 h minus admission GCS. Positive values indicated improvement, zero indicated no change, and negative values indicated deterioration. These measures were exploratory and did not convert the baseline admission GCS into a conventional longitudinal clinical outcome.

### 2.6. Hematologic, Iron-Status, Micronutrient, and Inflammatory Variables

Hemoglobin concentration was expressed in g/dL and analyzed as a continuous variable. Anemia was defined as a hemoglobin concentration below 13 g/dL in male patients and below 12 g/dL in female patients. RDW-CV was expressed as a percentage, and MCV was expressed in femtoliters.

Serum ferritin was expressed in ng/mL and analyzed as a continuous variable. Low ferritin was defined as a concentration below 30 ng/mL. Serum iron was expressed in µg/dL. Ferritin and serum iron were interpreted as complementary but non-equivalent indicators, as ferritin may vary in response to systemic inflammation.

Serum vitamin B12 was expressed in pg/mL and analyzed as a continuous variable. Low vitamin B12 status was defined as a concentration below 200 pg/mL. The database did not contain measurements of folate, homocysteine, methylmalonic acid, transferrin, total iron-binding capacity, or transferrin saturation; these parameters were therefore not inferred from the available data.

CRP was expressed in mg/L and analyzed as a continuous marker of systemic inflammation. It was treated as a nonspecific inflammatory biomarker and was not interpreted as evidence of any specific inflammatory source.

Continuous biomarker measurements constituted the principal biological exposure definitions. Anemia, ferritin below 30 ng/mL, and vitamin B12 below 200 pg/mL were retained as secondary pragmatic categorizations. Because these abnormalities were sparsely distributed across the ordered GCS categories and produced marked separation in the original threshold-based regression models, they were treated primarily as descriptive clinical characteristics in the revised analysis and were not interpreted through adjusted threshold-based ordinal odds ratios.

### 2.7. Covariates

Age, sex, and admission NIHSS were included as core adjustment variables in the principal single-biomarker ordinal regression models. Admission NIHSS was included because focal neurological deficit severity was assessed during the same pre-thrombolysis evaluation as GCS and differed across the ordered admission GCS categories. GCS and NIHSS were not considered interchangeable measures; rather, NIHSS adjustment was used to account for an important component of baseline stroke severity that may influence total GCS. Admission NIHSS was available for all 95 patients and was included as a core covariate in the principal age-, sex-, and NIHSS-adjusted analyses.

Residence, acute myocardial infarction, congestive heart failure, transient ischemic attack, previous stroke, diabetes mellitus, hypertension, atrial fibrillation, coronavirus disease 2019 (COVID-19), history of cancer, smoking, alcohol use, obesity, motor deficit, aphasia, and dysarthria were used for cohort characterization and unadjusted comparisons across admission GCS categories. Because congestive heart failure and atrial fibrillation showed the most prominent between-category differences among available comorbidities, targeted sensitivity analyses additionally evaluated each principal biomarker model after sequential adjustment for congestive heart failure and atrial fibrillation and after simultaneous inclusion of both variables. This limited strategy was selected to address prominent available confounding without fitting extensively parameterized models in the 95-patient cohort.

Clinician-documented aphasia was evaluated in a separate targeted sensitivity family because language impairment may influence the verbal component of total GCS. Aphasia was not independently adjudicated and was therefore not treated as a validated research-grade language measure.

In-hospital outcomes in this manuscript were limited to the length of stay, in-hospital death, and survival to discharge. The length of stay was recorded in days for the index hospitalization, while death denoted death before hospital discharge, and survival to discharge denoted patients alive at discharge. Hemorrhagic classifications, functional outcome scales, neurological deterioration endpoints, and bleeding outcomes were not analyzed as in-hospital outcomes in this manuscript.

Broad vascular territory was available for all 95 linked patients, but its distribution was highly imbalanced and therefore unsuitable for stable covariate adjustment in the baseline ordinal models. Routine-care reperfusion status was documented after the pre-thrombolysis admission GCS assessment and was therefore not treated as a confounder of baseline GCS; it was used only in an additional sensitivity analysis of the exploratory 24 h GCS change outcome. ASPECTS was available in only six linked records and was not modeled. Details regarding lesion location, infarct volume, collateral status, stroke etiology, standardized reperfusion adjudication, chronic kidney disease stage, active malignancy status, chronic inflammatory disease burden, active infection, and validated nutritional status were not available as uniform patient-level covariates. Onset-to-needle and door-to-needle values existed in a separate 95-record process table without a common patient identifier and were therefore not merged into the GCS analyses. Consequently, adjustment for NIHSS, aphasia, congestive heart failure, or atrial fibrillation should not be interpreted as establishing independence from unmeasured lesion location, infarct volume, or other unavailable stroke-related and systemic confounders.

### 2.8. Statistical Analysis

Analyses were performed using Python 3.13.5 with pandas 2.2.3, NumPy 2.3.5, SciPy 1.17.0, and statsmodels 0.14.6. Figures were generated using Matplotlib 3.10.8. Continuous variables were evaluated using histograms, quantile–quantile plots, summary statistics, and distributional tests. Approximately symmetric variables were summarized as means and standard deviations, whereas asymmetric variables were summarized as medians and interquartile ranges. Categorical variables were reported as absolute frequencies and percentages.

Characteristics were described for the complete cohort and across the ordered GCS categories. Continuous variables were compared using one-way analysis of variance when distributional and variance assumptions were satisfied, or with the Kruskal–Wallis test if otherwise.

Categorical variables were compared using the chi-square test when expected cell frequencies were adequate. Sparse 3 × 2 tables were evaluated using the Fisher–Freeman–Halton exact test, calculated by the complete enumeration of all tables with the observed margins.

Spearman rank correlation coefficients were calculated between the original four-level admission GCS scores and age, admission NIHSS, hemoglobin, RDW-CV, MCV, ferritin, serum iron, vitamin B12, and CRP. Ninety-five percent confidence intervals were estimated using the percentile method from a common set of 5000 patient-level nonparametric bootstrap resamples generated with a fixed random seed of 20260708. The same resampled patient indices were applied to every correlation. The complete correlation matrix was also used descriptively to examine cross-marker relationships among erythrocyte indices, iron-status markers, vitamin B12, and CRPs. No formal interaction term involving chronic kidney disease or nutritional status was fitted because these variables were not available as standardized patient-level measures.

Proportional odds ordinal logistic regression was used for the principal adjusted analyses. Each biomarker was evaluated in a separate principal model adjusted for age, sex, and admission NIHSS. For transparency and comparability with the initial analysis, age- and sex-adjusted models were retained as supportive models but were no longer designated as the principal inferential family.

Continuous predictors were standardized using the cohort means and standard deviations; the resulting odds ratios therefore represented the odds of belonging to a higher admission GCS category per one-standard-deviation increase in the investigated predictor. Ferritin and vitamin B12 were log-transformed because their observed distributions were right-skewed.

Because previous evidence and the observed data suggested a potentially nonlinear association, MCV was evaluated first as a linear continuous exposure and then with an additional quadratic term within the principal age-, sex-, and NIHSS-adjusted specification. The quadratic specification was retained when it improved the model fit by likelihood-ratio testing and produced interpretable predicted probabilities across the observed MCV range. Given the limited cohort size and restricted GCS distribution, the fitted curve was interpreted as evidence of an exploratory nonlinear association rather than as defining a precise clinical threshold. Threshold-defined anemia, ferritin below 30 ng/mL, and vitamin B12 below 200 pg/mL were summarized using counts and percentages across the ordered GCS categories. Sparse cell distributions and marked separation, particularly the absence of anemia in the GCS 14 category, limited the robustness of adjusted threshold-based ordinal regression. These threshold variables were therefore retained primarily as descriptive secondary findings rather than as a separate inferential regression family.

Biological-domain models included hemoglobin, RDW-CV, and MCV in the hematologic domain; log-transformed ferritin, serum iron, and log-transformed vitamin B12 in the iron-status and micronutrient domain; and CRP in the inflammatory domain. These age- and sex-adjusted models were retained as secondary exploratory analyses and were not part of the principal inferential family.

Within the hematologic domain, the quadratic MCV term was retained only when it improved model fit after simultaneous adjustment for the other hematologic variables. Pairwise correlations and variance inflation factors were examined to assess collinearity. Automated stepwise variable selection was not used. The biological-domain models were intended to explore attenuation among biologically related variables. Their *p* values were treated as nominal and were not included in the false discovery rate correction applied to the principal single-biomarker model family.

The proportional odds assumption was evaluated using a Brant-type coefficient-equality assessment. For reproducibility, the three-level ordered outcome was represented by its two cumulative binary thresholds in a stacked logistic model. The equality of the slope coefficients across thresholds was tested jointly using patient-clustered robust covariance without a small-sample correction. A *p* value below 0.05 was interpreted as evidence against the proportional odds assumption.

Model convergence, coefficient direction, standard errors, confidence-interval width, influential observations, and proportional odds diagnostics were examined for the principal models. Targeted cardiovascular sensitivity analyses added congestive heart failure and atrial fibrillation sequentially and then simultaneously to each principal single-biomarker model.

A separate sensitivity family additionally included clinician-documented aphasia. Exploratory analyses assessed absolute GCS at 24 h and a three-level change measure comprising deterioration, no change, and improvement. The 24 h ordered-change models were additionally repeated with indicators for documented complete and partial reperfusion, using patients with neither documented complete nor partial reperfusion as the reference category. Broad vascular territory was summarized descriptively rather than modeled because only three patients had vertebrobasilar territory and one was undetermined; ASPECTS was not modeled because values were available for only six patients.

For interpretation, the seven age-, sex-, and admission NIHSS-adjusted single-biomarker ordinal models constituted the principal inferential family. Unadjusted correlations and age- and sex-adjusted models were used for supportive analyses. The targeted NIHSS-plus-aphasia models and the models additionally accounting for congestive heart failure and atrial fibrillation were used for sensitivity analyses. Threshold-defined abnormalities were treated primarily as descriptive secondary findings because of sparse cell distributions and separations. Biological-domain and 24 h analyses remained either secondary or exploratory.

The false discovery rate control used the Benjamini–Hochberg procedure within the seven-biomarker principal family. For targeted sensitivity analyses, the aphasia-adjusted and reperfusion-adjusted 24 h change models each formed separate seven-biomarker families. Sequential CHF-only and atrial-fibrillation-only models were examined as robustness checks. For the combined CHF-plus-atrial-fibrillation sensitivity analysis, Benjamini–Hochberg adjustment was applied to the six biomarkers modeled with linear terms; MCV was evaluated separately using the nonlinear specification described above.

All statistical tests were two-sided. Interpretation prioritized effect estimates, 95% confidence intervals, false discovery rate-adjusted *p* values, and consistency across models.

The final dataset contained no missing values for the principal baseline neurological measure, principal biological exposures, age, sex, admission NIHSS, or exploratory 24 h GCS measures. No imputation was required. No prospective sample-size calculation or post hoc power calculation was performed, as the analysis included the entire fixed retrospective cohort.

### 2.9. Ethical Considerations

The protocol governing the retrospective use of existing medical records was reviewed and approved by the Ethics Committee of “Saint Andrew the Apostle” Clinical Emergency County Hospital, Galati, Romania, approval no. 14225, dated 6 July 2026. The approval was issued within the doctoral research project entitled “Management of the Geriatric Patient with Thrombolysis-Treated versus Non-Thrombolysis-Treated Acute Ischemic Stroke”. The clinical care evaluated in this study occurred between 2020 and 2024 and was completed before the research analysis. No treatment decisions, investigations, or patient-management procedures were performed for research purposes. Research-specific data extraction, pseudonymization, and statistical analysis were undertaken under the approved retrospective protocol. Direct patient identifiers were excluded from the analytical dataset.

The study was conducted in accordance with the Declaration of Helsinki and the applicable national and European requirements concerning the processing and protection of personal data.

## 3. Results

### 3.1. Baseline Cohort Characteristics

After the cohort derivation described in Section 2.2 and shown Figure 1, the final analytical cohort comprised 95 unique individuals, each represented by one retained index hospitalization.

All 95 patients had complete admission GCS and principal biological exposure data. The observed admission GCS range was 11–14, with no GCS 15 value in the retained analytical cohort; no patient had been excluded because of GCS magnitude.

The mean age of the cohort was 76.8 ± 6.9 years. The cohort included 54 male patients (56.8%) and 41 female patients (43.2%). Admission GCS scores were distributed as follows: two patients had a score of 11, 19 had a score of 12, 35 had a score of 13, and 39 had a score of 14. For ordinal analyses, 21 patients were included in the GCS 11–12 category, 35 in the GCS 13 category, and 39 in the GCS 14 category.

Baseline demographic, neurological, and clinical characteristics across the ordered admission GCS categories are summarized in Table 1.

Patients in the lower admission GCS categories were older and had higher admission NIHSS scores, with nominal between-category *p* values of 0.004 for each variable. Congestive heart failure, atrial fibrillation, and clinician-documented aphasia also showed nominal differences across the ordered GCS categories. Aphasia was documented in 11 of 21 patients with GCS 11–12, 20 of 35 with GCS 13, and 11 of 39 with GCS 14; these frequencies were not independently adjudicated for diagnostic misclassification.

Sex, residence, previous stroke, diabetes mellitus, hypertension, obesity, dysarthria, length of hospital stay, and in-hospital mortality did not show nominally significant between-category differences. Age, sex, and admission NIHSS were included as core adjustment variables in the principal single-biomarker models, as prespecified in the revised analytical hierarchy. Additional demographic, clinical, comorbidity, and behavioral characteristics are presented in Supplementary Table S1.

Patient-linked stroke-characterization data showed carotid-territory involvement in 91 patients (95.8%), vertebrobasilar involvement in three (3.2%), and an undetermined broad territory in one (1.1%). Routine-care reperfusion documentation classified 15 patients (15.8%) as complete reperfusion, 42 (44.2%) as partial reperfusion, and 38 (40.0%) as having neither documented complete nor partial reperfusion. ASPECTS was available in only six patients (6.3%) and was not analyzed inferentially. These distributions by admission GCS category are provided in Supplementary Table S7.

### 3.2. Biomarker Distributions Across Admission GCS Categories

All seven continuous biomarker comparisons remained statistically significant after false discovery rate (FDR) correction. Patients in the GCS 11–12 category had lower median hemoglobin, ferritin, serum iron, vitamin B12, and MCV values and higher median CRP and RDW-CV values than patients in the higher GCS categories.

Anemia was identified in 21 of the 95 patients (22.1%), ferritin below 30 ng/mL in 15 patients (15.8%), and vitamin B12 below 200 pg/mL in 22 patients (23.2%). Threshold-defined abnormalities were concentrated in the lower GCS category: anemia occurred in 17 out of 21 patients with GCS 11–12, as compared to four out of 35 with GCS 13 and zero out of 39 with GCS 14. Low ferritin and low vitamin B12 also occurred most frequently in GCS 11–12. These unadjusted distributions do not account for nutritional status, chronic inflammatory conditions, renal dysfunction, or active infection, which were not available as standardized confounders in the principal models.

The hematologic, iron-status, micronutrient, and inflammatory profiles according to admission GCS category are presented in Table 2.

### 3.3. Unadjusted Correlations with Admission GCS

Admission GCS showed positive monotonic correlations with hemoglobin, ferritin, serum iron, vitamin B12, and MCV and inverse correlations with CRP and RDW-CV. The strongest biomarker correlations were observed for RDW-CV (rho = −0.843, 95% bootstrap CI, −0.892 to −0.774) and MCV (rho = 0.779, 95% bootstrap CI, 0.668 to 0.866).

Age and admission NIHSS showed more modest inverse correlations with admission GCS. All seven biomarker correlations remained statistically significant after FDR correction, and their bootstrap confidence intervals excluded zero.

**Figure 2:**
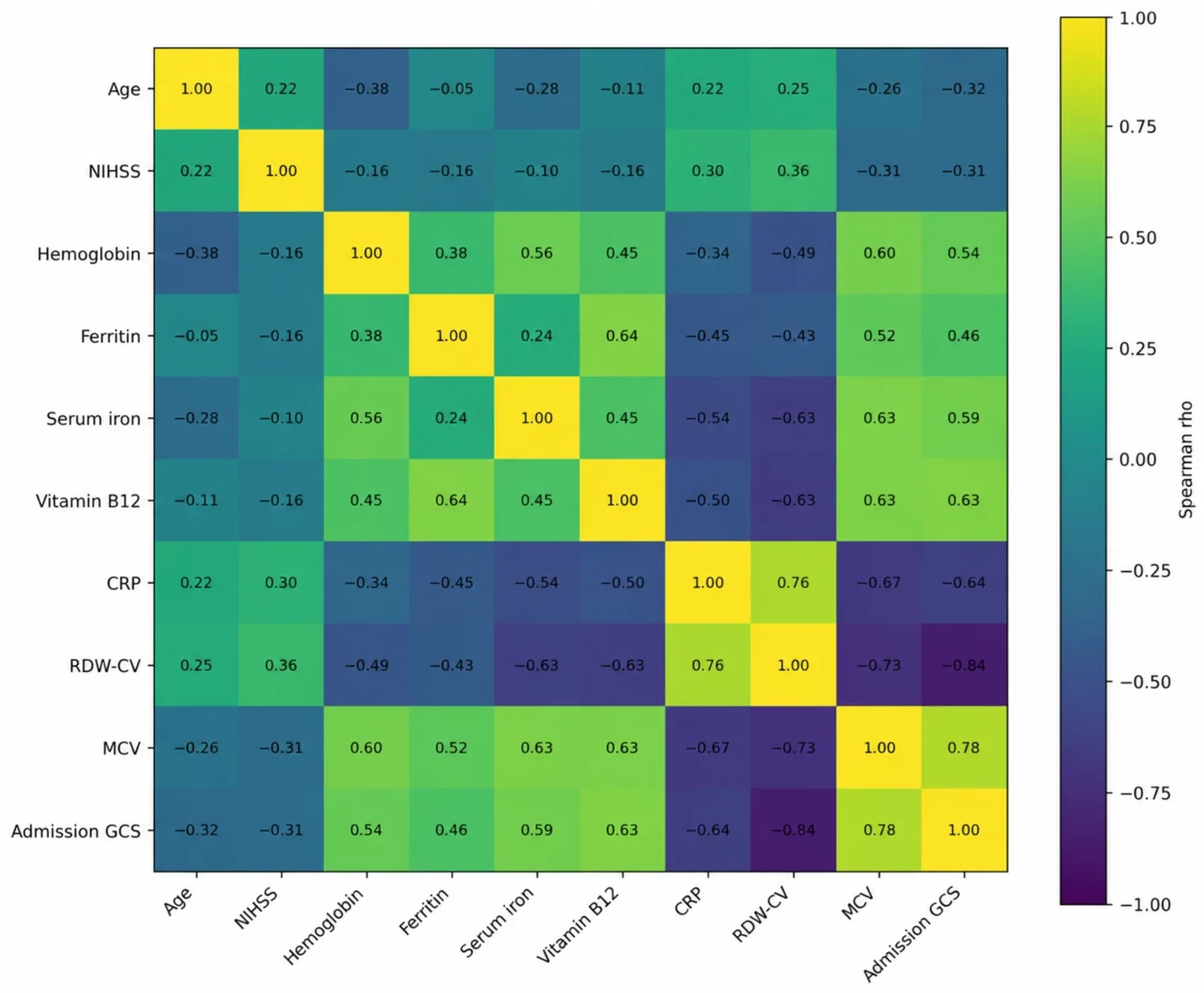
Spearman correlation matrix for admission GCS, age, admission NIHSS, and the investigated biological parameters. Positive coefficients indicate concordant rank relationships, whereas negative coefficients indicate inverse rank relationships. CRP, C-reactive protein; RDW-CV, red cell distribution width coefficient of variation; MCV, mean corpuscular volume; GCS, Glasgow Coma Scale; NIHSS, National Institutes of Health Stroke Scale.

The direction and magnitude of the relationships among admission GCS, neurological severity, age, and the investigated biomarkers are shown in Figure 2.

The correlation matrix also showed substantial cross-marker relationships relevant to biological interpretation. RDW-CV correlated positively with CRP (rho = 0.761) and inversely with ferritin (rho = −0.430), serum iron (rho = −0.630), vitamin B12 (rho = −0.634), and MCV (rho = −0.732). MCV correlated positively with ferritin (rho = 0.521), serum iron (rho = 0.630), and vitamin B12 (rho = 0.628) and inversely with CRP (rho = −0.669). These coefficients are descriptive cross-marker associations rather than evidence of causal interaction. The complete numerical matrix is provided in Supplementary Table S2.

The numerical correlation estimates and their bootstrap confidence intervals are presented in Table 3.

### 3.4. Principal Age-, Sex-, and Admission NIHSS-Adjusted Ordinal Regression

In the principal proportional odds models adjusted for age, sex, and admission NIHSS, higher hemoglobin, log-transformed ferritin, serum iron, and log-transformed vitamin B12 were associated with greater odds of belonging to a higher admission GCS category, whereas higher CRP and RDW-CV were associated with lower odds of belonging to a higher category. Because continuous predictors were standardized, the reported odds ratios represent the change in the odds of a higher admission GCS category per one-standard-deviation increase in each predictor.

The principal adjusted ORs were 5.68 for hemoglobin (95% CI, 2.92–11.05), 3.01 for log-transformed ferritin (95% CI, 1.78–5.09), 4.71 for serum iron (95% CI, 2.62–8.47), 4.71 for log-transformed vitamin B12 (95% CI, 2.27–9.78), 0.20 for CRP (95% CI, 0.11–0.36), and 0.019 for RDW-CV (95% CI, 0.005–0.077). All six associations remained statistically significant after FDR correction. The proportional odds assumption was not rejected for any of these principal single-biomarker models.

Age- and sex-adjusted models were retained as supportive analyses and yielded closely comparable association directions and magnitudes. In the targeted sensitivity family additionally adjusted for clinician-documented aphasia, the association directions remained unchanged, with FDR-adjusted *p* values below 0.001 for all seven biomarker specifications.

Targeted cardiovascular sensitivity analyses also accounted for congestive heart failure and atrial fibrillation, the two available comorbidities showing the most prominent between-category differences in Table 1. Sequential adjustment for each comorbidity and simultaneous adjustment for both did not alter the direction of the principal biomarker associations. In the combined models including age, sex, admission NIHSS, congestive heart failure, and atrial fibrillation, all six linear biomarker associations remained statistically significant after FDR correction. Complete estimates are reported in Supplementary Table S10.

**Figure 3:**
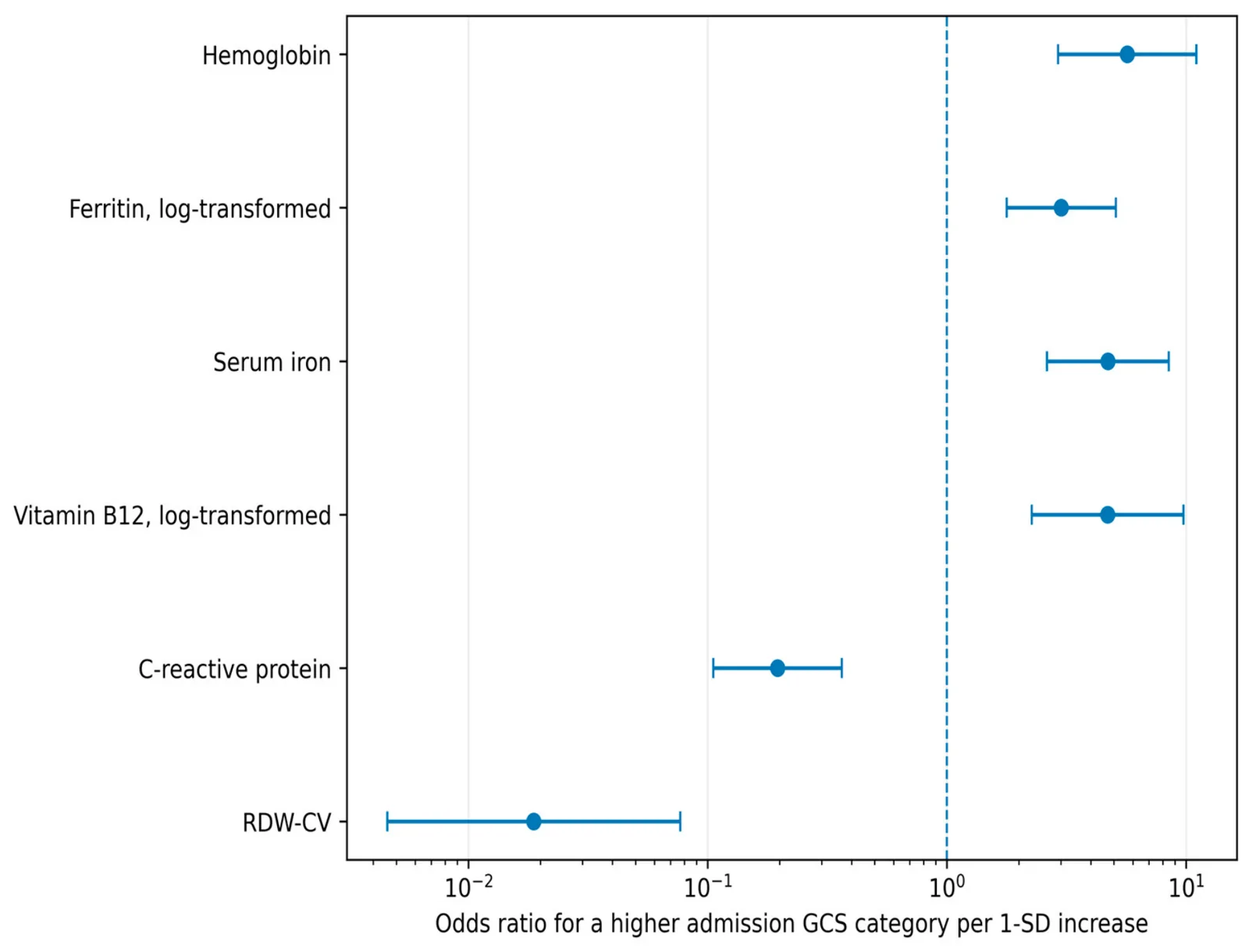
Principal age-, sex-, and admission NIHSS-adjusted proportional odds estimates for biomarkers modeled using linear predictor terms. Points represent adjusted odds ratios, and horizontal lines represent 95% confidence intervals. Odds ratios are expressed per one-standard-deviation increase in each predictor; ferritin and vitamin B12 were log-transformed before standardization. The vertical dashed line indicates the null value of 1, and the *x*-axis is displayed on a logarithmic scale. MCV is presented separately in Figure 4 because a quadratic specification provided a better fit within the principal age-, sex-, and NIHSS-adjusted model. OR, odds ratio; CI, confidence interval; CRP, C-reactive protein; RDW-CV, red cell distribution width coefficient of variation; NIHSS, National Institutes of Health Stroke Scale.

The principal age-, sex-, and admission NIHSS-adjusted estimates for biomarkers modeled with linear predictor terms are presented in Figure 3. MCV is addressed separately because the quadratic specification provided a better fit than the linear specification.

The complete estimates from the unadjusted, supportive age- and sex-adjusted, and principal age-, sex-, and admission NIHSS-adjusted models are presented in Table 4.

### 3.5. Exploratory Nonlinear Association Between MCV and Admission GCS

Within the principal age-, sex-, and admission NIHSS-adjusted specification, addition of a quadratic MCV term improved the model fit compared with the corresponding linear specification (likelihood-ratio χ^2^ = 10.30, df = 1, *p* = 0.001). The joint contribution of the linear and quadratic MCV terms was also statistically significant relative to the covariate-only model (likelihood-ratio χ^2^ = 76.06, df = 2, FDR-adjusted *p* < 0.001). The proportional odds assumption was not rejected for the quadratic model (*p* = 0.150).

**Figure 4:**
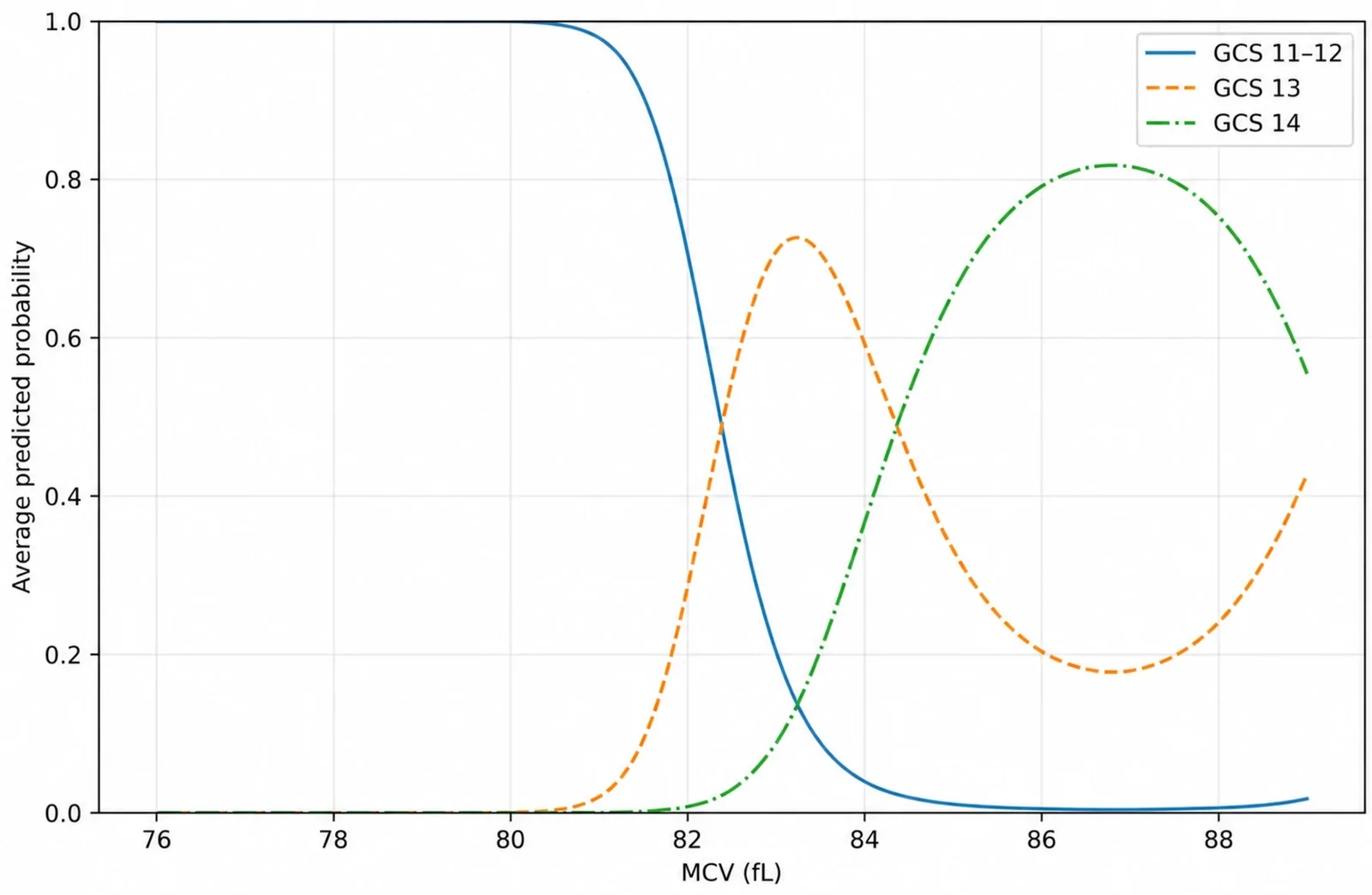
Exploratory average predicted probabilities of the three admission GCS categories across the observed MCV range, estimated from the quadratic proportional odds model adjusted for age, sex, and admission NIHSS. The solid line represents GCS 11–12, the dashed line represents GCS 13, and the dash–dot line represents GCS 14. The curves illustrate the fitted nonlinear association across the observed MCV range; no model-derived turning point or clinical cutoff is indicated. GCS, Glasgow Coma Scale; MCV, mean corpuscular volume; NIHSS, National Institutes of Health Stroke Scale.

The fitted relationship was therefore interpreted as an exploratory nonlinear association across the observed MCV range rather than as evidence for a precise biological or clinical threshold. Given the retrospective single-center design, sample size, and restricted admission GCS distribution, no exact model-derived turning point was emphasized. The preference for a quadratic rather than linear MCV specification also persisted in a targeted sensitivity model additionally accounting for congestive heart failure and atrial fibrillation (likelihood-ratio χ^2^ = 13.57, df = 1, *p* < 0.001). The average predicted probabilities of the three admission GCS categories across the observed MCV range, derived from the principal quadratic model, are shown in Figure 4.

Taken together, these findings support a nonlinear association between MCV and admission GCS across the observed range, without supporting the interpretation of any single MCV value as a clinical cutoff or treatment threshold.

### 3.6. Descriptive Threshold-Defined Clinical Findings

Threshold-defined abnormalities were strongly concentrated in the lower admission GCS categories. Anemia was present in 21 out of 95 patients overall and occurred in 17 out of 21 patients with GCS 11–12, four out of 35 with GCS 13, and none of the 39 patients with GCS 14. Ferritin below 30 ng/mL occurred in 15 patients overall, including nine out of 21 with GCS 11–12, three out of 35 with GCS 13, and three out of 39 with GCS 14. Vitamin B12 below 200 pg/mL occurred in 22 patients overall, including 16 out of 21 with GCS 11–12, five out of 35 with GCS 13, and one out of 39 with GCS 14. Because these threshold-defined variables showed sparse cell distributions and marked separation across the ordered GCS categories, particularly the absence of anemia among patients with GCS 14, adjusted threshold-based ordinal odds ratios were not retained as inferential results in the revised analysis. These variables are therefore presented primarily as descriptive clinical characteristics. Their distributions are summarized in Table 2 and Supplementary Table S4.

### 3.7. Secondary Biological-Domain Analyses

Secondary biological-domain models were retained to examine attenuation of biomarker associations after simultaneous consideration of biologically related parameters. These models were not part of the principal inferential family and remained adjusted for age and sex.

Within the hematologic domain, RDW-CV and the linear MCV term retained nominal associations with admission GCS category, whereas the hemoglobin association was attenuated after simultaneous inclusion of the three erythrocyte-related parameters. Within the iron-status and micronutrient domains, ferritin and serum iron retained nominal associations, whereas vitamin B12 did not remain statistically significant after mutual adjustment. CRP remained associated with admission GCS in the secondary inflammatory model.

These domain analyses are interpreted as exploratory assessments of overlapping biological information rather than as evidence of independent biomarker effects. Full estimates and diagnostics are provided in Supplementary Table S11.

### 3.8. Exploratory 24 h Analyses

At 24 h, 22 patients had a lower GCS score than at admission, 38 had no change, and 35 had a higher score. Pre-thrombolysis biomarkers remained associated with absolute GCS at 24 h in descriptive analyses; however, none was associated with the numerical or ordered direction of GCS change after FDR correction. The absence of an association with change was unchanged in sensitivity models additionally accounting for documented reperfusion status. In the reperfusion-adjusted ordered-change family, no biomarker remained statistically significant after FDR correction, with all FDR-adjusted *p* values ≥ 0.441. Complete estimates are reported in Supplementary Tables S6A,B and S8. Accordingly, the 24 h analyses did not provide evidence that the investigated baseline biomarkers were associated with the direction of early neurological change.

## 4. Discussion

### 4.1. Principal Findings

In this cohort of older adults with acute ischemic stroke treated with intravenous alteplase, the principal age-, sex-, and admission NIHSS-adjusted analyses identified a coordinated baseline biological pattern. Higher pre-thrombolysis hemoglobin, ferritin, serum iron, and vitamin B12 were associated with higher admission GCS categories, whereas higher CRP and RDW-CV were associated with lower categories. MCV showed an exploratory nonlinear association across the observed range.

The directions of the principal associations were retained in targeted sensitivity analyses that additionally accounted for clinician-documented aphasia and for congestive heart failure and atrial fibrillation. These sensitivity analyses support the stability of the observed directions with respect to the measured covariates included in those models, but they do not establish independence from unmeasured stroke characteristics or other residual confounders.

Threshold-defined anemia, low ferritin, and low vitamin B12 were concentrated in the lower GCS categories but were treated primarily as descriptive findings because sparse cell distributions and marked separation limited robust adjusted ordinal inference. Secondary biological-domain analyses indicated substantial overlap among hematologic, iron-status, micronutrient, and inflammatory information rather than distinct independent biomarker effects.

Importantly, no investigated baseline biomarker was associated with the direction of GCS change during the first 24 h after FDR correction, including models additionally accounting for documented reperfusion status. The principal findings therefore describe concurrent cross-sectional biological correlates of the baseline clinical state and should not be interpreted as evidence of an association with early neurological recovery.

### 4.2. Admission GCS as a Measure of Overall Clinical Responsiveness

Admission GCS was selected because the study examined concurrent pre-thrombolysis biological correlates of baseline overall responsiveness at emergency presentation, not because GCS was considered a conventional stroke outcome or a substitute for NIHSS. GCS and NIHSS assess overlapping but non-equivalent neurological constructs: NIHSS emphasizes focal neurological deficits, whereas GCS summarizes ocular, verbal, and motor responsiveness [1]. Admission NIHSS was therefore included in the principal adjusted models to account for an important component of baseline focal neurological severity.

Interpretations of the total GCS remain constrained in acute ischemic stroke. Aphasia, dysarthria, impaired comprehension, severe focal motor deficits, lesion location, and infarct volume may influence the total GCS without representing a pure alteration in arousal. Component-level GCS was unavailable, and clinician-documented aphasia was not independently adjudicated. Association directions remained similar in sensitivity models additionally accounting for aphasia, but this does not substitute for component-level GCS, standardized language assessment, or detailed lesion characterization.

Persistence of the observed associations after NIHSS and aphasia adjustment should not be interpreted as evidence that the biomarker–GCS relationships are independent of lesion location or infarct volume. Broad vascular territory was available, but 91 of 95 patients had carotid-territory involvement, leaving insufficient variation for stable adjustment. Detailed lesion location and infarct volume were unavailable.

The admission GCS distribution was also restricted to 11–14, with only two patients scoring 11 and no patient scoring 15. Accordingly, GCS in this study is best regarded as a baseline measure of overall clinical responsiveness within this specific alteplase-treated cohort, and the findings should not be extrapolated to the full GCS spectrum.

### 4.3. Hemoglobin, Anemia, and Erythrocyte Indices

Higher hemoglobin was associated with higher admission GCS categories in the principal age-, sex-, and NIHSS-adjusted analysis. A registry of 6866 thrombolysis-treated patients evaluated anemia in relation to three-month outcome, mortality, and symptomatic intracranial hemorrhage [10], while other studies reported nonlinear or inconsistent hemoglobin associations [3,4]. These outcomes differ from the baseline responsiveness measure examined here, limiting direct comparison.

Clinically defined anemia was strongly concentrated in the lower GCS categories, occurring in 17 out of 21 patients with GCS 11–12, four out of 35 with GCS 13, and none of the 39 patients with GCS 14. Because this distribution produced marked separation, anemia was retained as a descriptive clinical characteristic rather than interpreted through an adjusted threshold-based ordinal odds ratio.

RDW-CV remained strongly inversely associated with admission GCS in the principal adjusted analysis. A higher RDW has previously been associated with greater NIHSS-defined stroke severity and unfavorable outcome [5].

RDW-CV is not disease-specific and may reflect overlapping erythropoietic, nutritional, renal, and inflammatory influences. In the present cohort, RDW-CV correlated positively with CRP (rho = 0.761) and inversely with ferritin (rho = −0.430), serum iron (rho = −0.630), vitamin B12 (rho = −0.634), and MCV (rho = −0.732), indicating substantial biological overlap between erythrocyte heterogeneity, iron status, micronutrient status, and systemic inflammation. Iron restriction and inflammatory activity may both alter erythrocyte production and size variability, while impaired renal function may further influence erythropoiesis through reduced erythropoietin activity, inflammation, and disturbances in iron availability. Renal dysfunction and inflammatory markers have shown clinical relevance in thrombolysis-treated stroke populations [11,12], and RDW has also been associated with adverse renal outcomes in chronic kidney disease [13]. Because the kidney disease stage, detailed renal function characterization, chronic inflammatory disease activity, validated nutritional status, and longitudinal inflammatory measurements were not available as uniform patient-level covariates, these interrelated processes remain potential sources of residual confounding. Accordingly, the RDW-CV association should not be interpreted as representing an isolated erythrocyte effect or a single underlying biological pathway.

MCV showed an exploratory nonlinear association with admission GCS. A 2025 study likewise reported a nonlinear relationship between MCV and three-month functional outcome [6], although that outcome differs from the baseline neurological measure used here. In the present cohort, MCV correlated positively with ferritin (rho = 0.521), serum iron (rho = 0.630), and vitamin B12 (rho = 0.628) and inversely with CRP (rho = −0.669) and RDW-CV (rho = −0.732). Several biological processes could plausibly contribute to this nonlinear relationship. Acute physiological stress and inflammatory activity may transiently modify erythrocyte indices, whereas chronic nutritional deficiency, systemic disease, altered erythropoiesis, liver-related factors, medication exposure, or reticulocytosis may influence MCV through different pathways and over different time scales [14]. The coexistence of these acute and chronic influences could generate a non-monotonic pattern; however, because folate status, validated nutritional assessment, detailed liver-related variables, reticulocyte measurements, and longitudinal inflammatory data were unavailable, the observed association cannot be assigned to a specific pathophysiological process or interpreted as defining a biological or clinical threshold.

### 4.4. Iron Status and Ferritin

Higher serum iron and ferritin were associated with higher admission GCS categories in the principal age-, sex-, and NIHSS-adjusted models. A prospective multi-center cohort associated iron deficiency at admission with unfavorable 90-day outcome [7], but used ferritin together with transferrin saturation and therefore applied a more comprehensive definition of iron deficiency than was available in the present dataset. The current findings should therefore be interpreted as associations involving measured ferritin and serum iron rather than as evidence of etiologically confirmed nutritional iron deficiency.

Ferritin reflects iron stores but is also an acute-phase reactant, while circulating serum iron may decrease during inflammatory and infectious states. In the present correlation matrix, ferritin correlated inversely with CRP (rho = −0.445) and positively with vitamin B12 (rho = 0.641), whereas serum iron correlated inversely with CRP (rho = −0.539). Nutritional status, chronic inflammatory activity, renal dysfunction, malignancy, and active infection may therefore confound relationships among ferritin, serum iron, erythrocyte indices, and neurological status, reinforcing the biological overlap described above for RDW-CV. CRP and renal function have shown joint relevance in thrombolysis-treated acute ischemic stroke [12].

Standardized nutritional assessment, chronic inflammatory disease activity, active-infection status, and kidney disease staging were not incorporated as uniform covariates. Distinguishing absolute iron deficiency from inflammation-associated functional iron restriction would additionally require transferrin, total iron-binding capacity, transferrin saturation, and related iron-regulatory measures. Low ferritin below 30 ng/mL was therefore retained primarily as a descriptive threshold-defined characteristic.

### 4.5. Vitamin B12

Higher continuous vitamin B12 concentrations were associated with higher admission GCS categories in the principal adjusted model, consistent with previous reports relating lower vitamin B12 concentrations to stroke severity and functional outcome [8]. Vitamin B12 below 200 pg/mL was concentrated predominantly in the lower GCS categories but was retained as a descriptive threshold-defined characteristic because of sparse distribution across categories.

Vitamin B12 also correlated with ferritin, serum iron, RDW-CV, MCV, and CRP, indicating that its association occurred within a broader nutritional, hematologic, and inflammatory pattern. Without folate, homocysteine, methylmalonic acid, or holotranscobalamin measurements, serum vitamin B12 alone cannot fully characterize functional vitamin B12 status or establish a specific mechanistic pathway.

### 4.6. C-Reactive Protein and Systemic Inflammation

Higher CRP was associated with lower admission GCS categories in the principal age-, sex-, and NIHSS-adjusted model. Pre-thrombolysis high-sensitivity CRP has been associated with treatment response [11], and CRP added discriminatory information to a clinical model for in-hospital outcome in another thrombolysis-treated cohort [12]. Those studies examined different outcomes and therefore provide biological context rather than direct validation of the present baseline association.

CRP was the only inflammatory marker available and is nonspecific. Its concentration may reflect the acute ischemic response, pre-existing or low-grade inflammation, systemic or localized infection, vascular or metabolic disease, renal dysfunction, malignancy-related inflammation, or other systemic conditions. These processes may simultaneously influence hemoglobin, ferritin, serum iron, vitamin B12, RDW-CV, MCV, and neurological responsiveness. The present analyses therefore cannot distinguish a direct CRP-GCS relationship from residual confounding by shared inflammatory or systemic determinants.

### 4.7. Clinical Interpretation

The investigated biomarkers are available during routine emergency assessment, but the present models were designed to evaluate associations rather than clinical prediction. The study did not evaluate discrimination, calibration, reclassification, incremental predictive value, decision curve performance, or clinical utility. The reported estimates therefore should not be used to predict an individual patient’s GCS, determine thrombolysis eligibility, modify reperfusion decisions, or define biomarker-based treatment thresholds.

The stability of association directions after NIHSS, aphasia, congestive heart failure, and atrial fibrillation adjustment does not convert these relationships into causal or clinically actionable effects. Prospective model development and external validation would be required before any predictive application could be considered.

The absence of FDR-significant associations with the direction of 24 h GCS change further limits clinical interpretation. The observed biomarker relationships primarily characterize the biological profile accompanying the baseline neurological state and do not demonstrate an association with early neurological recovery after thrombolysis.

### 4.8. Strengths and Limitations

The study’s strengths include patient-level deduplication, complete data for the principal analyses, measurement of the investigated biomarkers before thrombolysis, use of the original four-level GCS scores for rank correlations and ordered categories for regression, bootstrap confidence intervals, FDR controls, proportional odds diagnostics, and principal adjustments for age, sex, and admission NIHSS. Targeted sensitivity analyses additionally examined clinician-documented aphasia and the prominent available cardiovascular comorbidities—congestive heart failure and atrial fibrillation. MCV nonlinearity was evaluated explicitly rather than assuming a uniform linear relationship.

The retrospective single-center design and fixed sample of 95 alteplase-treated patients limit generalizability and control of unmeasured confounding. Admission GCS ranged only from 11 to 14, with two, 19, 35, and 39 patients at scores 11, 12, 13, and 14, respectively; no patient had admission GCS 15. Combining GCS 11 and 12 avoided a two-patient ordinal stratum but did not remove the information limitation created by the narrow observed range. The estimates therefore apply only to the represented GCS categories and should not be extrapolated to the full GCS spectrum, non-thrombolyzed patients, or broader acute stroke populations.

Component-level GCS was unavailable, and total GCS may have been influenced by aphasia, dysarthria, impaired comprehension, and focal motor deficits. Broad vascular territory was available for all 95 linked patients but was highly imbalanced, with 91 carotid-territory cases, three vertebrobasilar cases, and one undetermined case. ASPECTS was available in only six records.

This limited ASPECTS availability precluded meaningful adjustment for imaging-defined baseline ischemic burden and represents an important limitation of stroke-severity characterization. By contrast, admission NIHSS was available for all 95 patients and was included in the principal adjusted models as a standardized measure of baseline neurological deficit severity. NIHSS adjustment, however, cannot be a substitute for detailed lesion location, infarct volume, collateral status, or systematic imaging-based severity assessments. The archive also did not permit uniform determination of imaging modality, direct imaging confirmation of acute infarction in every case, or standardized TICI/mTICI reperfusion grading.

Detailed lesion location, infarct volume, standardized imaging-modality metadata, collateral circulation, stroke etiology, and standardized angiographic reperfusion adjudication were unavailable. Consequently, persistence of the biomarker associations after NIHSS and aphasia adjustment does not demonstrate independence from lesion location or infarct volume.

Routine-care reperfusion status was available and was incorporated into sensitivity analyses of 24 h GCS change, but reperfusion occurred after the baseline admission assessment and was therefore not an appropriate confounder of admission GCS. The reperfusion-adjusted 24 h models remained null after FDR correction. Onset-to-needle and door-to-needle data were retained in a separate 95-record process table without a common patient identifier and could not be linked safely to the analytical cohort.

Mechanical thrombectomy status and endovascular-treatment eligibility criteria were not retained as uniform linkable patient-level variables. This limits characterization of concomitant reperfusion pathways and precludes a reliable cohort-level thrombectomy proportion; however, all 95 patients in the analytical cohort were documented as having received intravenous alteplase.

Congestive heart failure and atrial fibrillation were available and were therefore examined in targeted sensitivity analyses. These analyses did not materially alter the directions of the principal associations. However, the cohort size did not support unrestricted adjustment for all recorded comorbidities simultaneously, and residual confounding remains possible. Chronic kidney disease stage, active malignancy status, chronic inflammatory disease activity, active infection, and validated nutritional status were not available as uniform patient-level covariates.

Biological parameters were measured once before thrombolysis. Serial trajectories and folate, homocysteine, methylmalonic acid, transferrin, total iron-binding capacity, transferrin saturation, hepcidin, and other mechanistic measurements were unavailable. Long-term functional outcome, recurrent stroke, post-discharge mortality, and 90-day modified Rankin Scale were also not recorded.

Several biomarker associations yielded large, standardized odds ratios within the restricted admission GCS range. These effect magnitudes require cautious interpretation given the cohort size (n = 95), the sparse lowest GCS stratum, and the marked concentration of adverse biomarker values in the lower GCS categories. This concern is particularly relevant to RDW-CV, for which the principal adjusted odds ratio was 0.019 per one-standard-deviation increase. The extreme numerical magnitude of this estimate may partly reflect strong between-category separation within the present sample and may therefore overstate the magnitude that would be expected in a larger or more heterogeneous population. Similar caution applies to the other large standardized continuous biomarker odds ratios. Although model convergence, confidence-interval estimation, proportional odds diagnostics, and targeted sensitivity analyses supported the direction of these associations, they cannot establish the stability of their exact effect magnitudes. Threshold-defined abnormalities were therefore retained primarily as descriptive findings because of sparse-data sensitivity, while the principal continuous biomarker odds ratios should be interpreted as exploratory within-cohort association estimates rather than as stable population effect sizes.

### 4.9. Research Implications

Prospective multi-center studies should evaluate broader admission GCS distributions and include separate eye, verbal, and motor GCS components, standardized language assessments, NIHSS, detailed lesion locations and infarct volumes, standardized neuroimaging, collateral status, reperfusion success, treatment time metrics, renal function, active infection, chronic inflammatory conditions, nutritional status, relevant comorbidities, and 90-day functional and survival outcomes.

Serial hematologic, inflammatory, iron-status, and micronutrient measurements would help distinguish persistent biological characteristics from acute-phase changes. The observed MCV nonlinearity should be evaluated using prespecified flexible models in independent cohorts rather than interpreted as defining a fixed biological threshold.

Future prediction research should be conducted separately from association analyses and should include independent evaluation of discrimination, calibration, incremental values or reclassification where appropriate, and clinical utility before any clinical application.

If oral-systemic hypotheses are investigated in future stroke cohorts, standardized oral and periodontal assessments should be incorporated prospectively. Such hypotheses cannot be evaluated from the present dataset because oral and periodontal status were not measured.

## 5. Conclusions

In this single-center retrospective cohort of 95 adults aged 65 years or older with acute ischemic stroke treated with intravenous alteplase and an observed admission GCS range of 11–14, higher pre-thrombolysis hemoglobin, ferritin, serum iron, and vitamin B12 and lower CRP and RDW-CV were associated with higher ordered admission GCS categories in principal models adjusted for age, sex, and admission NIHSS. MCV showed an exploratory nonlinear association across the observed range. Association directions remained consistent in targeted sensitivity analyses that additionally accounted for clinician-documented aphasia and for congestive heart failure and atrial fibrillation.

These findings represent concurrent cross-sectional biological correlates of the baseline clinical state. Persistence after measured-covariate adjustment does not establish independence from lesion location, infarct volume, or other unmeasured stroke-related and systemic factors. No biomarker was associated with the direction of 24 h GCS change after FDR correction, including analyses additionally accounting for documented reperfusion status; the present results therefore do not demonstrate an association with early neurological recovery.

The findings are associative and should not be interpreted as causal effects, validated predictors, or biomarker-based treatment thresholds. Their generalizability is constrained by the single-center design, restricted GCS distribution, sample size, and residual confounding. Prospective multi-center validation with broader neurological characterization, detailed imaging, longitudinal outcomes, and more complete biological confounder measurements is required.

## Figures and Tables

**Table 1:** Demographic, clinical, and neurological characteristics according to admission GCS category.

Characteristic	Overall N = 95	GCS 11–12 n = 21	GCS 13 n = 35	GCS 14 n = 39	*p* Value
Age, years	76.8 ± 6.9	79.8 ± 7.7	78.0 ± 6.8	74.1 ± 5.6	0.004
Admission NIHSS	12.9 ± 4.6	15.5 ± 5.3	12.9 ± 4.1	11.4 ± 4.2	0.004
Length of stay, days	10.0 [6.0–12.5]	11.0 [5.0–18.0]	10.0 [7.0–12.5]	9.0 [5.5–12.0]	0.365
Male sex	54 (56.8%)	9 (42.9%)	21 (60.0%)	24 (61.5%)	0.338
Female sex	41 (43.2%)	12 (57.1%)	14 (40.0%)	15 (38.5%)	—
Rural residence	32 (33.7%)	10 (47.6%)	12 (34.3%)	10 (25.6%)	0.228
Congestive heart failure	31 (32.6%)	10 (47.6%)	17 (48.6%)	4 (10.3%)	<0.001
Previous stroke	12 (12.6%)	3 (14.3%)	6 (17.1%)	3 (7.7%)	0.453
Diabetes mellitus	29 (30.5%)	7 (33.3%)	12 (34.3%)	10 (25.6%)	0.687
Hypertension	80 (84.2%)	17 (81.0%)	30 (85.7%)	33 (84.6%)	0.876
Atrial fibrillation	35 (36.8%)	12 (57.1%)	17 (48.6%)	6 (15.4%)	0.001
Obesity	19 (20.0%)	5 (23.8%)	7 (20.0%)	7 (17.9%)	0.897
Aphasia	42 (44.2%)	11 (52.4%)	20 (57.1%)	11 (28.2%)	0.030
Dysarthria	54 (56.8%)	10 (47.6%)	18 (51.4%)	26 (66.7%)	0.262
In-hospital death	25 (26.3%)	7 (33.3%)	12 (34.3%)	6 (15.4%)	0.130

**Note:** Continuous variables are presented as mean ± standard deviation or median [interquartile range], and categorical variables as n (%). Group comparisons were performed using one-way analysis of variance, the Kruskal–Wallis test, the chi-square test, or the Fisher–Freeman–Halton exact test according to variable distribution and expected cell frequencies. For the complementary categories of male and female sex, the *p* value is reported once in the first row. These comparisons were descriptive, and the reported *p* values were not adjusted for multiple testing. Aphasia reflects the treating neurologist’s routine-care admission documentation and was not independently adjudicated with a research-specific language battery.

**Table 2:** Hematologic, iron-status, micronutrient, and inflammatory parameters according to admission GCS category.

Biomarker	Overall N = 95	GCS 11–12 n = 21	GCS 13 n = 35	GCS 14 n = 39	Nominal *p*	FDR-Adjusted *p*
Hemoglobin, g/dL	13.9 [12.6–15.0]	11.6 [11.3–12.6]	14.0 [13.8–14.7]	14.7 [13.7–15.7]	<0.001	<0.001
Ferritin, ng/mL	53.0 [36.0–62.0]	30.0 [28.0–36.0]	52.0 [40.5–60.0]	58.0 [51.5–66.0]	<0.001	<0.001
Serum iron, µg/dL	52.0 [44.0–58.0]	38.0 [34.0–42.0]	50.0 [46.5–52.5]	57.0 [52.0–60.0]	<0.001	<0.001
Vitamin B12, pg/mL	220.0 [205.0–235.0]	185.0 [180.0–190.0]	215.0 [205.0–225.0]	235.0 [225.0–240.0]	<0.001	<0.001
CRP, mg/L	28.0 [22.0–32.5]	36.0 [32.0–40.0]	28.0 [26.0–32.0]	22.0 [20.0–26.0]	<0.001	<0.001
RDW-CV, %	16.2 [15.8–17.0]	17.3 [17.1–17.8]	16.4 [16.1–16.9]	15.7 [15.6–15.9]	<0.001	<0.001
MCV, fL	84.0 [83.0–85.0]	82.0 [81.0–82.0]	84.0 [83.0–84.0]	85.0 [85.0–86.0]	<0.001	<0.001
Anemia	21 (22.1%)	17 (81.0%)	4 (11.4%)	0 (0.0%)	—	—
Ferritin < 30 ng/mL	15 (15.8%)	9 (42.9%)	3 (8.6%)	3 (7.7%)	—	—
Vitamin B12 < 200 pg/mL	22 (23.2%)	16 (76.2%)	5 (14.3%)	1 (2.6%)	—	—

**Note:** Continuous variables are presented as median [interquartile range], and categorical variables as n (%). Comparisons across the ordered admission GCS categories were performed using the Kruskal–Wallis test for continuous variables. False discovery rate adjustment was performed using the Benjamini–Hochberg procedure for the family of seven continuous biomarkers. Threshold-defined anemia, ferritin below 30 ng/mL, and vitamin B12 below 200 pg/mL are presented descriptively because sparse cell distributions and marked separation limited robust adjusted ordinal inference; no inferential *p* values are reported for these three threshold-defined variables. FDR, false discovery rate; CRP, C-reactive protein; RDW-CV, red cell distribution width coefficient of variation; MCV, mean corpuscular volume.

**Table 3:** Spearman correlations with the original four-level admission GCS scores.

Variable	Spearman Rho	95% Bootstrap CI	Nominal *p*	FDR-Adjusted *p*
Age	−0.320	−0.492 to −0.126	0.002	Not adjusted
Admission NIHSS	−0.312	−0.495 to −0.111	0.002	Not adjusted
Hemoglobin	0.545	0.371 to 0.688	<0.001	<0.001
Ferritin	0.459	0.258 to 0.628	<0.001	<0.001
Serum iron	0.595	0.420 to 0.741	<0.001	<0.001
Vitamin B12	0.634	0.466 to 0.769	<0.001	<0.001
C-reactive protein	−0.638	−0.775 to −0.470	<0.001	<0.001
RDW-CV	−0.843	−0.892 to −0.774	<0.001	<0.001
MCV	0.779	0.668 to 0.866	<0.001	<0.001

**Note:** Spearman rho coefficients were calculated using the original four-level admission GCS scores. Confidence intervals were estimated using 5000 patient-level nonparametric bootstrap resamples. Ferritin and vitamin B12 are presented on their original clinical scales because Spearman rank correlations were invariant under monotonic log transformation. False discovery rate adjustment using the Benjamini–Hochberg procedure was applied only to the family of seven biomarker correlations; age and admission NIHSS were not included in this correction. CI, confidence interval; RDW-CV, red cell distribution width coefficient of variation; MCV, mean corpuscular volume.

**Table 4:** Ordinal logistic regression models for admission GCS category.

Biomarker	Unadjusted OR (95% CI)	Supportive Age- and Sex-Adjusted OR (95% CI)	Principal Age-, Sex-, and NIHSS-Adjusted OR (95% CI)	FDR-Adjusted *p*, Principal Model	PO Test *p*, Principal Model
Hemoglobin	5.00 (2.78–9.00)	5.15 (2.69–9.86)	5.68 (2.92–11.05)	<0.001	0.117
Ferritin, log-transformed	3.07 (1.81–5.19)	3.17 (1.87–5.38)	3.01 (1.78–5.09)	<0.001	0.385
Serum iron	4.94 (2.79–8.75)	4.49 (2.53–7.99)	4.71 (2.62–8.47)	<0.001	0.130
Vitamin B12, log-transformed	5.04 (2.47–10.26)	4.90 (2.34–10.26)	4.71 (2.27–9.78)	<0.001	0.555
C-reactive protein	0.18 (0.098–0.32)	0.18 (0.096–0.33)	0.20 (0.11–0.36)	<0.001	0.625
RDW-CV	0.019 (0.005–0.072)	0.018 (0.004–0.071)	0.019 (0.005–0.077)	<0.001	0.545

**Note:** Odds ratios represent the change in the odds of belonging to a higher admission GCS category per one-standard-deviation increase in the investigated predictor. The age-, sex-, and admission NIHSS-adjusted models constituted the principal inferential family. Age- and sex-adjusted models were retained as supportive analyses. Ferritin and vitamin B12 were log-transformed before standardization. FDR adjustment using the Benjamini–Hochberg procedure was applied to the seven-biomarker principal family, with MCV evaluated using the nonlinear specification described in Section 3.5. The PO test *p* value refers to the corresponding principal age-, sex-, and admission NIHSS-adjusted model. MCV is not included in this table because the quadratic specification provided a better fit than the linear specification. OR, odds ratio; CI, confidence interval; FDR, false discovery rate; PO, proportional odds; NIHSS, National Institutes of Health Stroke Scale; RDW-CV, red cell distribution width coefficient of variation.

## Data Availability

The data supporting the findings of this study are not publicly available as they were derived from hospital medical records and are subject to patient confidentiality, ethical restrictions, and institutional data protection requirements. Requests for data or analyses codes should be directed to the corresponding authors and will be considered subject to the required institutional and ethical approvals.

## Supplementary Materials

The following supporting information can be downloaded at: https://www.mdpi.com/article/10.3390/medicina62091692/s1, Table S1: Complete demographic and clinical characteristics by admission GCS category; Table S2: Complete Spearman correlation matrix; Table S3: Proportional odds assumption diagnostics for the principal age-, sex-, and admission NIHSS-adjusted models; Table S4: Descriptive distribution of threshold-defined clinical characteristics by admission GCS category; Table S5: MCV model specification and sensitivity checks; Table S6A: Exploratory correlations with GCS at 24 hours and 24-hour GCS change ; Table S6B: Exploratory proportional odds models for the direction of GCS change at 24 h; Table S7: Available stroke-characterization variables by admission GCS category; Table S8: Exploratory proportional odds models for the direction of GCS change at 24 h, additionally adjusted for documented reperfusion status; Table S9: Targeted admission GCS sensitivity models, additionally adjusted for clinician-documented aphasia; Table S10: Targeted cardiovascular sensitivity analyses, additionally accounting for congestive heart failure and atrial fibrillation; Table S11: Secondary age- and sex-adjusted biological-domain proportional odds models for admission GCS categories.

## Abbreviations

The following abbreviations are used in this manuscript:

CI	Confidence interval
COVID-19	Coronavirus disease 2019
CRP	C-reactive protein
FDR	False discovery rate
GCS	Glasgow Coma Scale
IQR	Interquartile range
MCV	Mean corpuscular volume
NIHSS	National Institutes of Health Stroke Scale
OR	Odds ratio
PO	Proportional odds
RDW-CV	Red cell distribution width coefficient of variation
SD	Standard deviation
STROBE	Strengthening the Reporting of Observational Studies in Epidemiology
VIF	Variance inflation factor

## References

[1] Li J., Wang D., Tao W., Dong W., Zhang J., Yang J., Liu M. (2016). Early consciousness disorder in acute ischemic stroke: Incidence, risk factors and outcome. BMC Neurol..

[2] Bautista A.F., Lenhardt R., Yang D., Yu C., Heine M.F., Mascha E.J., Heine C., Neyer T.M., Remmel K., Akca O. (2019). Early prediction of prognosis in elderly acute stroke patients. Crit. Care Explor..

[3] Zhang R., Xu Q., Wang A., Jiang Y., Meng X., Zhou M., Wang Y., Liu G. (2021). Hemoglobin concentration and clinical outcomes after acute ischemic stroke or transient ischemic attack. J. Am. Heart Assoc..

[4] Sharma K., Johnson D.J., Johnson B., Frank S.M., Stevens R.D. (2018). Hemoglobin concentration does not impact 3-month outcome following acute ischemic stroke. BMC Neurol..

[5] Xue J., Zhang D., Zhang X.G., Zhu X.Q., Xu X.S., Yue Y.H. (2022). Red cell distribution width is associated with stroke severity and unfavorable functional outcomes in ischemic stroke. Front. Neurol..

[6] Lv Z., Miao Y., Zhu M., Li F. (2025). Association between mean corpuscular volume and 3 month outcome in patients with acute ischemic stroke: A second analysis based on a prospective cohort study. Front. Neurol..

[7] Ciacciarelli A., Falcou A., Nicolini E., Broccolini A., Frisullo G., Abruzzese S., Scala I., Anticoli S., Testani E., Montinaro E. (2025). The prognostic role of iron deficiency in acute ischemic stroke patients: A prospective multicentric cohort study. J. Neurol. Sci..

[8] Atam V., Srivastava S., Sharma A., Atam I., Tewari J., Qidwai K.A. (2024). Serum vitamin B12 levels as a risk factor and prognostic marker in patients with acute ischemic stroke at a tertiary care center in Northern India: A case-control study. Cureus.

[9] Jiang J., Tan C., Zhou W., Peng W., Zhou X., Du J., Wang H., Mo L., Liu X., Chen L. (2021). Plasma C-reactive protein level and outcome of acute ischemic stroke patients treated by intravenous thrombolysis: A systematic review and meta-analysis. Eur. Neurol..

[10] Altersberger V.L., Kellert L., Al Sultan A.S., Martinez-Majander N., Hametner C., Eskandari A., Heldner M.R., van den Berg S.A., Zini A., Padjen V. (2020). Effect of haemoglobin levels on outcome in intravenous thrombolysis-treated stroke patients. Eur. Stroke J..

[11] Cheng X.D., Wang D.Z., Zhang Q., Wang J.H., Li B.H., Zhang X., Zhang J., Zhou S., Jia L.J., Wang L.R. (2023). Predictive role of pre-thrombolytic hs-CRP on the safety and efficacy of intravenous thrombolysis in acute ischemic stroke. BMC Neurol..

[12] Huang Z., Zhu X., Xu X., Wang Y., Zhu Y., Chen D., Cao Y., Zhang X. (2024). The joint effects of inflammation and renal function status on in-hospital outcomes in patients with acute ischemic stroke treated with intravenous thrombolysis. BMC Neurol..

[13] Deng X., Gao B., Wang F., Zhao M., Wang J., Zhang L. (2022). Red Blood Cell Distribution Width Is Associated with Adverse Kidney Outcomes in Patients With Chronic Kidney Disease. Front. Med..

[14] Nagao T., Hirokawa M. (2017). Diagnosis and treatment of macrocytic anemias in adults. J. Gen. Fam. Med..

