# Supplementary material for: Pre-Thrombolysis Biological Correlates of Admission Glasgow Coma Scale in Older Adults with Acute Ischemic Stroke: A Retrospective Single-Center Cohort Study
Source: Medicina (Kaunas). 2026 Sep 3;62(9):1692. doi: 10.3390/medicina62091692 (PMC13609183; doi:10.3390/medicina62091692)
Supplement: Supplementary file 1 [file medicina-62-01692-s001.zip › medicina-4495784-supplementary.pdf]

Supplementary Material

Pre-Thrombolysis Biological Correlates of Admission Glasgow Coma Scale in Older Adults with Acute Ischemic Stroke: A Retrospective Single-Center Cohort Study

The supplementary analyses used the same cohort of 95 unique patients and the same variable definitions as the main manuscript. They provide extended demographic and clinical characteristics, the complete correlation matrix, proportional-odds diagnostics for the principal age-, sex-, and admission NIHSS-adjusted models, descriptive threshold-defined clinical characteristics, model-specification and MCV sensitivity analyses, exploratory 24-hour GCS analyses, available stroke-characterization data, and targeted sensitivity analyses accounting for clinician-documented aphasia and prominent cardiovascular comorbidities. The retained dataset contained total GCS scores but not separate eye, verbal, and motor components; component-level GCS analyses were therefore not possible. Aphasia was a clinician-documented routine-care variable and was not independently adjudicated with a research-specific language battery. A concordance audit verified patient-level linkage between the principal analytical dataset and the stroke-characterization table for all 95 patients using Study_ID and source record number. Sparse threshold-defined abnormalities were presented descriptively in the revised analysis because marked separation limited robust adjusted ordinal inference. Sparse 3 × 2 categorical comparisons in the baseline descriptive table used the fully enumerated Fisher-Freeman-Halton exact test.

Table S1. Complete demographic and clinical characteristics by admission GCS category.

Characteristic	Overall N=95	GCS 11–12 n=21	GCS 13 n=35	GCS 14 n=39	p value	Test
Age, years	76.8 ± 6.9	79.8 ± 7.7	78.0 ± 6.8	74.1 ± 5.6	0.004	One–way ANOVA
Admission NIHSS	12.9 ± 4.6	15.5 ± 5.3	12.9 ± 4.1	11.4 ± 4.2	0.004	One–way ANOVA
Length of stay, days	10.0 [6.0–12.5]	11.0 [5.0–18.0]	10.0 [7.0–12.5]	9.0 [5.5–12.0]	0.365	Kruskal–Wallis
Male sex	54 (56.8%)	9 (42.9%)	21 (60.0%)	24 (61.5%)	0.338	Chi–square
Female sex	41 (43.2%)	12 (57.1%)	14 (40.0%)	15 (38.5%)	—	Chi–square
Rural residence	32 (33.7%)	10 (47.6%)	12 (34.3%)	10 (25.6%)	0.228	Chi–square
Acute myocardial infarction	9 (9.5%)	3 (14.3%)	5 (14.3%)	1 (2.6%)	0.117	Fisher–Freeman–Halton exact
Congestive heart failure	31 (32.6%)	10 (47.6%)	17 (48.6%)	4 (10.3%)	<0.001	Chi–square
Transient ischemic attack	3 (3.2%)	2 (9.5%)	1 (2.9%)	0 (0.0%)	0.110	Fisher–Freeman–Halton exact
Previous stroke	12 (12.6%)	3 (14.3%)	6 (17.1%)	3 (7.7%)	0.453	Fisher–Freeman–Halton exact
Diabetes mellitus	29 (30.5%)	7 (33.3%)	12 (34.3%)	10 (25.6%)	0.687	Chi–square
Hypertension	80 (84.2%)	17 (81.0%)	30 (85.7%)	33 (84.6%)	0.876	Fisher–Freeman–Halton exact
Atrial fibrillation	35 (36.8%)	12 (57.1%)	17 (48.6%)	6 (15.4%)	0.001	Chi–square
COVID–19	11 (11.6%)	3 (14.3%)	3 (8.6%)	5 (12.8%)	0.774	Fisher–Freeman–Halton exact
History of cancer	7 (7.4%)	2 (9.5%)	2 (5.7%)	3 (7.7%)	0.886	Fisher–Freeman–Halton exact
Smoking	7 (7.4%)	2 (9.5%)	1 (2.9%)	4 (10.3%)	0.524	Fisher–Freeman–Halton exact
Alcohol use	15 (15.8%)	0 (0.0%)	7 (20.0%)	8 (20.5%)	0.057	Fisher–Freeman–Halton exact
Obesity	19 (20.0%)	5 (23.8%)	7 (20.0%)	7 (17.9%)	0.897	Fisher–Freeman–Halton exact
Motor deficit	92 (96.8%)	20 (95.2%)	35 (100.0%)	37 (94.9%)	0.438	Fisher–Freeman–Halton exact
Aphasia	42 (44.2%)	11 (52.4%)	20 (57.1%)	11 (28.2%)	0.030	Chi–square
Dysarthria	54 (56.8%)	10 (47.6%)	18 (51.4%)	26 (66.7%)	0.262	Chi–square
In–hospital death	25 (26.3%)	7 (33.3%)	12 (34.3%)	6 (15.4%)	0.130	Chi–square

** **Note:** Values are presented as mean ± standard deviation, median [interquartile range], or n (%). Group comparisons were performed using one–way analysis of variance, the Kruskal–Wallis test, the chi–square test, or the Fisher–Freeman–Halton exact test, according to variable distribution and expected cell*

frequencies. For the complementary categories of male and female sex, the p value is reported once on the male row. Aphasia reflects the treating neurologist's routine-care admission documentation and was not independently adjudicated.

Table S2. Complete Spearman correlation matrix.

Variable	Age	Admission NIHSS	Hemoglobin	Ferritin	Serum iron	Vitamin B12	CRP	RDW-CV	MCV	Admission GCS
**Age**	1.000	0.217	−0.382	−0.054	−0.275	−0.106	0.220	0.248	−0.265	−0.320
**Admission NIHSS**	0.217	1.000	−0.161	−0.165	−0.101	−0.156	0.300	0.364	−0.309	−0.312
**Hemoglobin**	−0.382	−0.161	1.000	0.380	0.560	0.446	−0.343	−0.495	0.596	0.545
**Ferritin**	−0.054	−0.165	0.380	1.000	0.239	0.641	−0.445	−0.430	0.521	0.459
**Serum iron**	−0.275	−0.101	0.560	0.239	1.000	0.445	−0.539	−0.630	0.630	0.595
**Vitamin B12**	−0.106	−0.156	0.446	0.641	0.445	1.000	−0.500	−0.634	0.628	0.634
**CRP**	0.220	0.300	−0.343	−0.445	−0.539	−0.500	1.000	0.761	−0.669	−0.638
**RDW-CV**	0.248	0.364	−0.495	−0.430	−0.630	−0.634	0.761	1.000	−0.732	−0.843
**MCV**	−0.265	−0.309	0.596	0.521	0.630	0.628	−0.669	−0.732	1.000	0.779
**Admission GCS**	−0.320	−0.312	0.545	0.459	0.595	0.634	−0.638	−0.843	0.779	1.000

* ***Note:*** *Values are Spearman rho coefficients calculated using the original continuous or ordinal variable values. Admission GCS was analyzed on its original four-level scale.*

Table S3. Proportional-odds assumption diagnostics for the principal age-, sex-, and admission NIHSS-adjusted models.

Model	Joint PO test p	Decision at alpha=0.05
Hemoglobin	0.117	Supported
Ferritin, log-transformed	0.385	Supported
Serum iron	0.130	Supported
Vitamin B12, log-transformed	0.555	Supported
CRP	0.625	Supported
RDW-CV	0.545	Supported
MCV quadratic model	0.150	Supported

* *Note: All models included age, sex, and admission NIHSS. The MCV row refers to the retained quadratic specification rather than the linear sensitivity specification. A p value below 0.05 was interpreted as evidence against the proportional-odds assumption. PO, proportional odds; NIHSS, National Institutes of Health Stroke Scale.*

Table S4. Descriptive distribution of threshold-defined clinical characteristics by admission GCS category.

Clinical characteristic	Overall N=95	GCS 11–12 n=21	GCS 13 n=35	GCS 14 n=39
Anemia	21 (22.1%)	17 (81.0%)	4 (11.4%)	0 (0.0%)
Ferritin <30 ng/mL	15 (15.8%)	9 (42.9%)	3 (8.6%)	3 (7.7%)
Vitamin B12 <200 pg/mL	22 (23.2%)	16 (76.2%)	5 (14.3%)	1 (2.6%)

* ***Note:*** *Threshold-defined abnormalities are presented descriptively. Marked imbalance and sparse cells across the ordered GCS categories, particularly the absence of anemia in the GCS 14 category, limited robust adjusted ordinal inference and produced separation in the original threshold-based regression models. Adjusted threshold-based odds ratios were therefore not retained as inferential results in the revised analysis.*

Table S5. MCV model-specification and sensitivity checks.

Diagnostic item	Model/test	Result	Interpretation
Principal MCV nonlinearity	Quadratic vs linear LR test, age + sex + NIHSS	LR χ^2 = 10.30; p = 0.001	Quadratic specification provided better fit
Principal MCV PO diagnostic	Quadratic age + sex + NIHSS model	PO p = 0.150	PO assumption supported
Overall MCV contribution	Linear + quadratic terms vs covariate-only model	LR χ^2 = 76.06; df = 2; FDR-adjusted p < 0.001	Overall MCV association supported within the principal family

Diagnostic item	Model/test	Result	Interpretation
Cardiovascular sensitivity	Quadratic vs linear LR test after additional CHF + AF adjustment	LR $\chi^2 = 13.57$; $p < 0.001$	Evidence of nonlinearity persisted
Cardiovascular PO diagnostic	Quadratic age + sex + NIHSS + CHF + AF model	PO p = 0.153	PO assumption supported

* ***Note:*** *No exact numerical location of the fitted directional change in MCV is reported because the nonlinear pattern was derived from a single retrospective cohort with a restricted GCS range and was not intended to define a clinical threshold. LR, likelihood ratio; PO, proportional odds; NIHSS, National Institutes of Health Stroke Scale; CHF, congestive heart failure; AF, atrial fibrillation; MCV, mean corpuscular volume; FDR, false discovery rate.*

Table S6A. Exploratory correlations with GCS at 24 hours and 24–hour GCS change.

Outcome	Biomarker	Spearman rho	Nominal p	FDR–adjusted p
GCS at 24 h	Hemoglobin	0.373	<0.001	<0.001
GCS at 24 h	Ferritin	0.260	0.011	0.011
GCS at 24 h	Serum iron	0.444	<0.001	<0.001
GCS at 24 h	Vitamin B12	0.315	0.002	0.002
GCS at 24 h	CRP	–0.415	<0.001	<0.001
GCS at 24 h	RDW–CV	–0.514	<0.001	<0.001
GCS at 24 h	MCV	0.469	<0.001	<0.001
GCS change at 24 h	Hemoglobin	0.056	0.587	0.938
GCS change at 24 h	Ferritin	–0.008	0.938	0.938
GCS change at 24 h	Serum iron	0.115	0.265	0.938
GCS change at 24 h	Vitamin B12	–0.072	0.486	0.938
GCS change at 24 h	CRP	–0.051	0.622	0.938
GCS change at 24 h	RDW–CV	–0.012	0.911	0.938
GCS change at 24 h	MCV	0.019	0.858	0.938

* ***Note:*** *GCS change was calculated as GCS at 24 hours minus admission GCS. False discovery rate adjustment using the Benjamini–Hochberg procedure was applied separately to the seven correlations with GCS at 24 hours and to the seven correlations with 24-hour GCS change. These exploratory analyses did not identify a baseline biomarker associated with the direction of early GCS change after FDR correction.*

Table S6B. Exploratory proportional-odds models for the direction of GCS change at 24 hours.

Biomarker	Adjusted OR for a higher change category	95% CI	Nominal p	FDR–adjusted p
Hemoglobin	1.57	0.93–2.66	0.090	0.490
Ferritin, log-transformed	0.96	0.64–1.45	0.857	0.857
Serum iron	1.35	0.84–2.18	0.217	0.490
Vitamin B12, log-transformed	1.26	0.77–2.07	0.355	0.497
CRP	0.75	0.47–1.22	0.247	0.490
RDW–CV	0.77	0.38–1.57	0.474	0.553
MCV	1.36	0.78–2.40	0.280	0.490

* ***Note:*** *The ordered outcome comprised deterioration, no change, and improvement. Models were adjusted for age, sex, and admission GCS. Odds ratios represent the change in the odds of belonging to a higher ordered change category per one-standard-deviation increase in the predictor. False discovery rate adjustment was applied to the family of seven biomarker models.*

Table S7. Available stroke-characterization variables by admission GCS category.

Characteristic	Overall N=95	GCS 11-12 n=21	GCS 13 n=35	GCS 14 n=39
Carotid territory	91 (95.8%)	20 (95.2%)	34 (97.1%)	37 (94.9%)
Vertebrobasilar territory	3 (3.2%)	1 (4.8%)	1 (2.9%)	1 (2.6%)
Undetermined broad vascular territory	1 (1.1%)	0 (0.0%)	0 (0.0%)	1 (2.6%)
Documented complete reperfusion	15 (15.8%)	2 (9.5%)	4 (11.4%)	9 (23.1%)
Documented partial reperfusion	42 (44.2%)	8 (38.1%)	15 (42.9%)	19 (48.7%)

Characteristic	Overall N=95	GCS 11-12 n=21	GCS 13 n=35	GCS 14 n=39
Neither documented complete nor partial reperfusion	38 (40.0%)	11 (52.4%)	16 (45.7%)	11 (28.2%)
ASPECTS available	6 (6.3%)	1 (4.8%)	2 (5.7%)	3 (7.7%)

* ***Note:*** *Vascular-territory and reperfusion fields were obtained from the patient-linked stroke-characterization table after concordance of Study_ID and source record number was verified for all 95 patients. The vascular-territory variables were broad routine-care labels (carotid, vertebrobasilar, or undetermined); detailed vessel-level or anatomical assignment criteria were not retained, and the categories were not retrospectively reclassified. Reperfusion was documented after the baseline admission GCS assessment and was therefore not treated as a confounder of admission GCS. Complete and partial reperfusion categories reflected routine-care documentation; no uniform TICI/mTICI grade or standardized patient-level angiographic adjudication rule defining complete versus partial reperfusion was retained. Mechanical thrombectomy status and endovascular-treatment eligibility criteria were not available as uniform linkable patient-level fields in the analytical archive. ASPECTS was available in only six records and was not analyzed inferentially. Admission NIHSS was available for all 95 patients and is reported in Table S1. No p values are reported because these stroke-characterization data are presented descriptively and several categories are sparse or post-baseline.*

Table S8. Exploratory proportional-odds models for the direction of GCS change at 24 hours additionally adjusted for documented reperfusion status.

Biomarker	Adjusted OR for a higher change category	95% CI	Nominal p	FDR-adjusted p
Hemoglobin	1.56	0.91-2.67	0.105	0.441
Ferritin, log-transformed	0.95	0.63-1.43	0.805	0.805
Serum iron	1.33	0.82-2.16	0.240	0.441
Vitamin B12, log-transformed	1.24	0.76-2.03	0.382	0.535
CRP	0.75	0.46-1.22	0.240	0.441
RDW-CV	0.77	0.38-1.58	0.478	0.558
MCV	1.40	0.79-2.49	0.252	0.441

* ***Note:*** *The ordered outcome comprised deterioration, no change, and improvement. Models were adjusted for age, sex, admission GCS, documented complete reperfusion, and documented partial reperfusion; patients with neither documented complete nor partial reperfusion formed the reperfusion reference category. Odds ratios represent the change in the odds of belonging to a higher ordered change category per one-standard-deviation increase in the biomarker. Ferritin and vitamin B12 were log-transformed before standardization. Benjamini-Hochberg adjustment was applied to the seven biomarker models. No biomarker remained statistically significant after FDR correction.*

Table S9. Targeted admission GCS sensitivity models additionally adjusted for clinician-documented aphasia.

Biomarker	Adjusted OR	95% CI	Nominal p	FDR-adjusted p
Hemoglobin	5.72	2.91-11.25	<0.001	<0.001
Ferritin, log-transformed	3.03	1.79-5.15	<0.001	<0.001
Serum iron	4.72	2.61-8.53	<0.001	<0.001
Vitamin B12, log-transformed	4.64	2.22-9.67	<0.001	<0.001
CRP	0.20	0.11-0.37	<0.001	<0.001
RDW-CV	0.019	0.005-0.077	<0.001	<0.001
MCV, linear sensitivity specification	18.22	6.55-50.72	<0.001	<0.001

* ***Note:*** *Each model included age, sex, admission NIHSS, clinician-documented aphasia, and one biomarker. Odds ratios represent the change in the odds of belonging to a higher admission GCS category per one-standard-deviation increase in the biomarker. Ferritin and vitamin B12 were log-transformed before standardization. MCV is shown only as a linear sensitivity specification and is not used as the principal MCV effect estimate because the quadratic specification provided better fit in the principal analysis. Benjamini–Hochberg adjustment was applied to this seven-biomarker sensitivity family. Aphasia was a routine-care clinician-documented variable and was not independently adjudicated.*

Table S10. Targeted cardiovascular sensitivity analyses additionally accounting for congestive heart failure and atrial fibrillation.

Biomarker	Principal OR (95% CI)	+ CHF OR (95% CI)	+ AF OR (95% CI)	+ CHF + AF OR (95% CI)	FDR-adjusted p, combined model
Hemoglobin	5.68 (2.92–11.05)	5.87 (2.95–11.70)	5.88 (3.01–11.50)	5.94 (3.00–11.73)	<0.001
Ferritin, log-transformed	3.01 (1.78–5.09)	3.84 (2.10–7.02)	3.18 (1.81–5.59)	3.89 (2.11–7.18)	<0.001
Serum iron	4.71 (2.62–8.47)	5.34 (2.89–9.86)	4.64 (2.60–8.28)	5.32 (2.88–9.83)	<0.001
Vitamin B12, log-transformed	4.71 (2.27–9.78)	5.47 (2.55–11.70)	5.16 (2.41–11.00)	5.61 (2.58–12.20)	<0.001
CRP	0.20 (0.106–0.365)	0.166 (0.083–0.330)	0.207 (0.112–0.385)	0.165 (0.082–0.332)	<0.001
RDW-CV	0.019 (0.00459–0.0769)	0.0091 (0.00153–0.0542)	0.0189 (0.00454–0.0782)	0.0067 (0.00096–0.0468)	<0.001

* ***Note:*** *The principal models included age, sex, admission NIHSS, and one biomarker. Sequential sensitivity models additionally included congestive heart failure (CHF) or atrial fibrillation (AF), and the combined sensitivity model*

included both CHF and AF. Odds ratios represent the change in the odds of belonging to a higher admission GCS category per one-standard-deviation increase in the biomarker. Ferritin and vitamin B12 were log-transformed before standardization. Benjamini-Hochberg adjustment was applied to the six linear-biomarker combined CHF-plus-AF sensitivity models. Association directions remained consistent across the sequential and combined cardiovascular adjustments. MCV was evaluated separately using its nonlinear specification; quadratic model preference persisted after additional CHF and AF adjustment (Table S5).

Table S11. Secondary age- and sex-adjusted biological-domain proportional-odds models for admission GCS category.

Model	Variable	Adjusted OR	95% CI	Nominal p value	VIF
Hematologic	Hemoglobin, per 1 SD	2.15	0.83–5.57	0.116	2.61
Hematologic	RDW-CV, per 1 SD	0.026	0.006–0.12	<0.001	2.66
Hematologic	MCV, per 1 SD, linear specification	4.84	1.39–16.77	0.013	3.58
Iron and micronutrient	Ferritin, log-transformed, per 1 SD	2.37	1.25–4.48	0.008	1.28
Iron and micronutrient	Serum iron, per 1 SD	3.56	1.96–6.47	<0.001	1.37
Iron and micronutrient	Vitamin B12, log-transformed, per 1 SD	1.46	0.64–3.31	0.367	1.35
Inflammatory	CRP, per 1 SD	0.18	0.096–0.33	<0.001	1.07

** **Note:** These biological-domain models were retained as secondary exploratory analyses and were not part of the principal inferential family. All models included age and sex. Ferritin and vitamin B12 were log-transformed before standardization. Within the hematologic domain, addition of a quadratic MCV term did not improve model fit after simultaneous adjustment for hemoglobin and RDW-CV (likelihood-ratio p = 0.489), and the linear MCV specification was therefore retained. Joint proportional-odds tests supported the hematologic (p = 0.413), iron and micronutrient (p = 0.063), and inflammatory (p = 0.678) domain models. The reported p values are nominal and were not adjusted for multiple testing. These models were used to explore attenuation among biologically related variables and should not be interpreted as establishing independent biomarker effects.*
